# Sophoridine Counteracts Obesity via Src-Mediated Inhibition of VEGFR Expression and PI3K/AKT Phosphorylation

**DOI:** 10.3390/ijms25021206

**Published:** 2024-01-19

**Authors:** Jingchun Sun, Xiaoting Wang, Yulin He, Xuekai Tian, Tiantian Yuan, Gongshe Yang, Taiyong Yu

**Affiliations:** Key Laboratory of Animal Genetics, Breeding and Reproduction of Shaanxi Province, Laboratory of Animal Fat Deposition & Muscle Development, College of Animal Science and Technology, Northwest A&F University, Yangling 712100, China; sunjc7497@nwafu.edu.cn (J.S.); nicexiaoting@163.com (X.W.); heyulin@nwafu.edu.cn (Y.H.); tianxuekai190@nwsuaf.edu.cn (X.T.); yuantiantian@nwafu.edu.cn (T.Y.); gsyang999@hotmail.com (G.Y.)

**Keywords:** sophoridine, obesity, Src, VEGFR2, PI3K/AKT, phosphorylation

## Abstract

Sophoridine (SRP) is a natural quinolizidine alkaloid found in many traditional Chinese herbs, though its effect on adipose tissue is unclear. We improved serum lipid levels by administering SRP by gavage in high-fat diet (HFD)-fed C57BL/6 mice. After 11 weeks, SRP supplementation significantly reduced body weight gain and improved glucose homeostasis, while reducing subcutaneous fat and liver weight. SRP also inhibited cell proliferation and differentiation of 3T3-L1 cells. Proteomics analysis revealed that SRP inhibits adipocyte differentiation by interacting with Src, thereby suppressing vascular endothelial growth factor receptor 2 (VEGFR2) expression and PI3K/AKT phosphorylation. This study provides an empirical basis for the treatment of obesity with small molecules.

## 1. Introduction

Obesity has become a global public health problem. The worldwide prevalence of obesity nearly tripled between 1975 and 2016 [1]. It is a major risk factor for cardiovascular diseases, type 2 diabetes, fatty liver, and several cancers, thereby contributing to a decline in both quality of life and life expectancy [2]. It is very important to prevent and treat obesity. FDA-approved drugs for long-term weight management include orlistat, lorcaserin, liraglutide, phentermine/topiramate, and naltrexone/bupropion [3], although these drugs are associated with serious gastrointestinal, central nervous system, and cardiovascular side effects [4,5]. Therefore, the search for effective, safe, and nontoxic weight loss substitutes are current areas of intense interest and active investigation.

In recent years, new natural compounds from plants have been investigated for their ability to prevent or treat obesity while also producing low toxicity, such as apigenin [6], capsaicin [7], and camptothecin [8]. Sophoridine (SRP) is a natural quinolizidine alkaloid isolated from traditional Chinese herbs such as the stems and leaves of the leguminous plant *Sophora alopecuroides* L., *Euchresta japonica Benth.*, and the roots of *S. alopecuroides Ait* [9]. Many studies have confirmed the pharmacological effects of SRP, including its anticancer [10], anti-inflammatory [11], and antiviral activities [12]. It has also been shown that matrine, an isomer of SRP, provides obesity resistance in mice by inducing adipose thermogenesis via activation of the *HSF1/PGC-1α* axis [13]. Therefore, we sought to determine whether SRP also binds Src to modulate its activity.

The Src kinase family includes non-receptor tyrosine kinases in three subfamilies. The SrcA subfamily includes Src, Yes, Fyn, and Fgr; the SrcB subfamily includes Lck, Hck, Blk, and Lyn; and Frk, Srm, and Brk comprise a third subfamily [14]. Src serves as a target, modulating fundamental physical activities including cell proliferation, apoptosis, adhesion, invasion, and movement via several signaling pathways including *Ras/STAT3/MAPK/PI3K/AKT* [15,16,17]. Src regulates the expression of angiogenic growth factor *VEGF* [18] and phosphorylates lipin-1 to promote glycerolipid synthesis [19].

In this study, C57BL/6J mice were fed a high-fat diet (HFD) and given an oral gavage of 20 mg/kg SRP daily for 11 weeks to explore the mechanisms of SRP on resistance to HFD-induced obesity, specifically via Src-PI3K/Akt signaling.

## 2. Results

### 2.1. SRP Provides Resistance to Diet-Induced Obesity and Metabolic Disorders in HFD-Fed Mice

**Figure 1:**
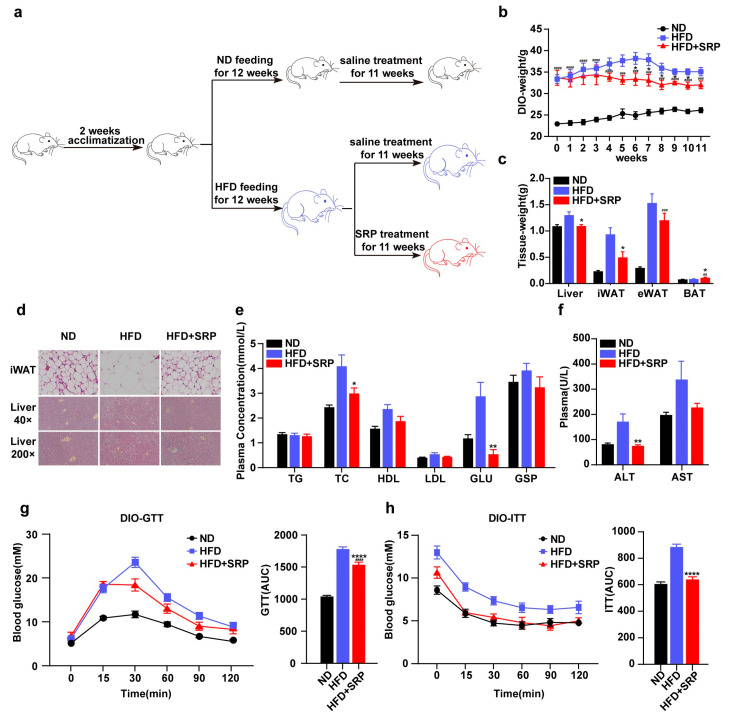
Sophoridine inhibits high-fat diet-induced bodyweight gain and improves glucose tolerance and insulin sensitivity in mice. (**a**) Schematic diagram of the animal experiment. The dose of SRP fed by gavage is 20 mg/kg. (**b**) Bodyweight curve during sophoridine treatment. (**c**) Tissue weight of liver, iWAT, eWAT, and BAT. (**d**) HE staining images of iWAT (scale bar = 10 μm) and liver (40×, 200×). (**e**) Plasma levels of triglyceride (TG), total cholesterol (TC), HDL, LDL, GLU, and GSP after 11 weeks treatment. (**f**) Plasma levels of ALT and AST. (**g**) Glucose tolerance test (GTT) and AUC. (**h**) Insulin tolerance test (ITT) and AUC. Data are presented as means ± SEM (*n* = 6–10). Statistical significance was analyzed by one-way ANOVA followed by Tukey’s post hoc tests. * *p* < 0.05, ** *p* < 0.01, **** *p* < 0.0001, vs. HFD mice; ## *p* < 0.01, ### *p* < 0.001, #### *p* < 0.0001, vs. ND mice. BAT: brown adipose tissue, iWAT: inguinal white adipose tissue, eWAT: epididymal white adipose tissue, TG: triglyceride, TC: total cholesterol, HDL: high density lipoprotein, LDL: low density lipoprotein, GLU: glucose, GSP: glycosylated serum protein.

We explored the role of SRP in a mouse model of HFD-induced obesity (Figure 1a). SRP gavage feeding treatment to the sixth week, bodyweights were significantly lower in the HFD-SRP group than in the untreated HFD group (Figure 1b). Among them, the weight of liver and iWAT was significantly lower than that of the HFD group (Figure 1c). Compared with the HFD group, adipocytes in the HFD-SRP group were smaller in size and had a greater number of adipose cells in the same microscopic field of view. In the liver tissue, HFD mice had larger lipid vacuoles, while the number of lipid vacuoles was significantly lower in the HFD-SRP mice (Figure 1d). Meanwhile, SRP significantly reduced the HFC-induced increases in plasma levels of total cholesterol (TC), glucose (Glu), and ALT (Figure 1e,f). SRP-treated mice showed better glucose tolerance and improved insulin sensitivity (Figure 1g,h). These results suggested that SRP reduced body weight and improved metabolic disorders.

### 2.2. SRP Inhibits 3T3-L1 Cell Proliferation

**Figure 2:**
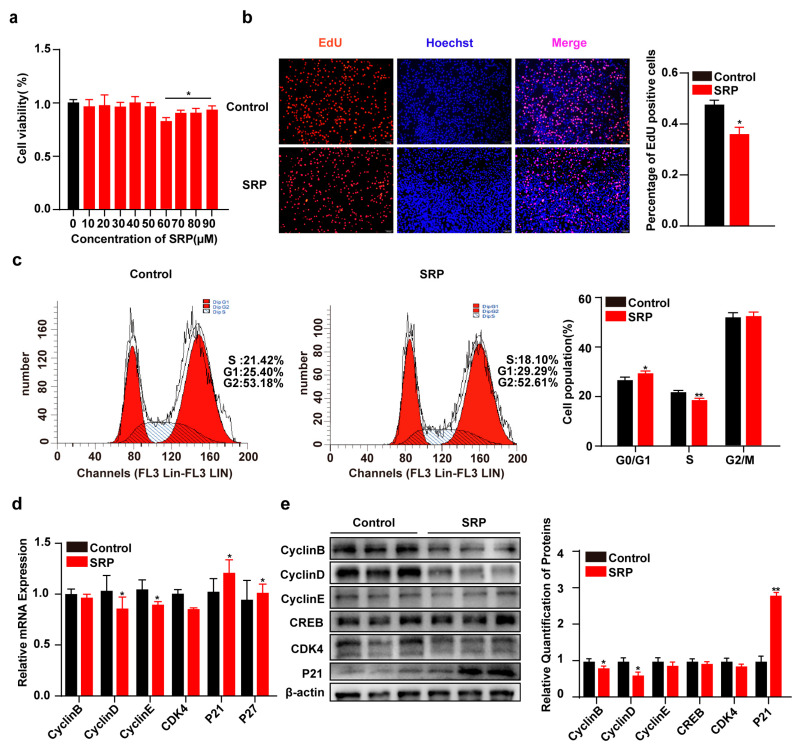
Sophoridine inhibits DNA replication and proliferation in 3T3-L1 cells. (**a**) 3T3-L1 cells were treated with sophoridine (10, 20, 30, 40, 50, 60, 70, 80, and 90 μM) and cell viability was measured. (**b**) EdU staining after treatment with sophoridine (50 μM) and quantitative analysis (scale bar = 100 μm). (**c**) The number of cells in different cell cycles was measured by flow cytometry. (**d**) Relative mRNA expression of proliferation marker genes cyclin B, cyclin D, cyclin E, CDK4, p21, and p27. (**e**) Western blot detection and quantitation of cell cycle marker gene expression. Data represent mean ± SEM (*n* = 3). Statistical analysis was performed by Student’s *t*-test, * *p* < 0.05, and ** *p* < 0.01.

To assess the effect of SRP on cell viability, 3T3-L1 cells were treated with different concentrations of SRP. Cell viability was significantly decreased at SRP concentrations above 50 μM, indicating that 50 μM SRP was not toxic to 3T3-L1 cells (Figure 2a). Therefore, 3T3-L1 cells were treated with 50 μM SRP to investigate its effect on proliferation. SRP significantly reduced the percentage of EdU-positive cells (Figure 2b). Flow cytometry results showed that SPR significantly reduced the population of cells in the S phase (Figure 2c) and transcript and protein levels of proliferation-related genes (Figure 2d,e).

### 2.3. SRP Inhibits the Differentiation of 3T3-L1 Cells

**Figure 3:**
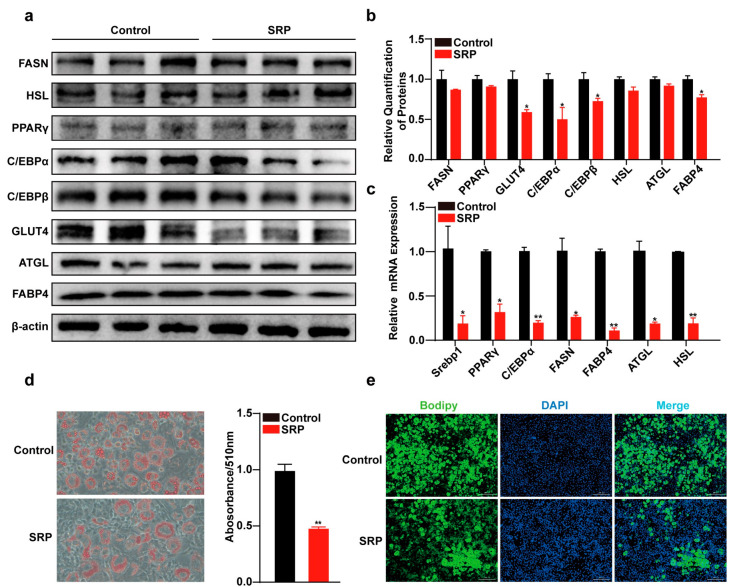
Sophoridine inhibits adipogenesis of 3T3-L1 cells. (**a**) Protein expression of adipogenesis-related FASN, PPARγ, C/EBPα, C/EBPβ, GLUT4, and FABP4 and lipolytic HSL and ATGL. (**b**) Quantitative analysis of Western blot. (**c**) Relative mRNA expression of adipogenesis-related and lipolytic genes. (**d**) Oil red O staining of 3T3-L1 cells differentiated at 6 days (200×), and determination of TG content. (**e**) Bodipy staining of 3T3-L1 cells (scale bar = 300 μm). Data represent mean ± SEM (*n* = 3). Statistical analysis was performed by Student’s *t*-test, * *p* < 0.05, and ** *p* < 0.01.

The effect of 50 μM SRP on 3T3-L1 differentiation was assessed. Bodipy staining of adipocytes on day 8 of differentiation revealed a significant decrease in the number of lipid droplets after SRP treatment (Figure 3e). Oil red O staining results were concordant (Figure 3d). In addition, the transcription of lipid synthesis-related genes, *SREBP1*, *PPARγ*, *C/EBPα*, *C/EBPβ*, and lipolysis-related genes, *ATGL* and *HSL*, was significantly reduced (*p* < 0.05) (Figure 3c). SRP significantly downregulated C/EBPα, C/EBPβ, GLUT4, and FABP4 protein expression (Figure 3a,b).

### 2.4. Proteomic Analysis of Differentially Expressed Proteins

**Figure 4:**
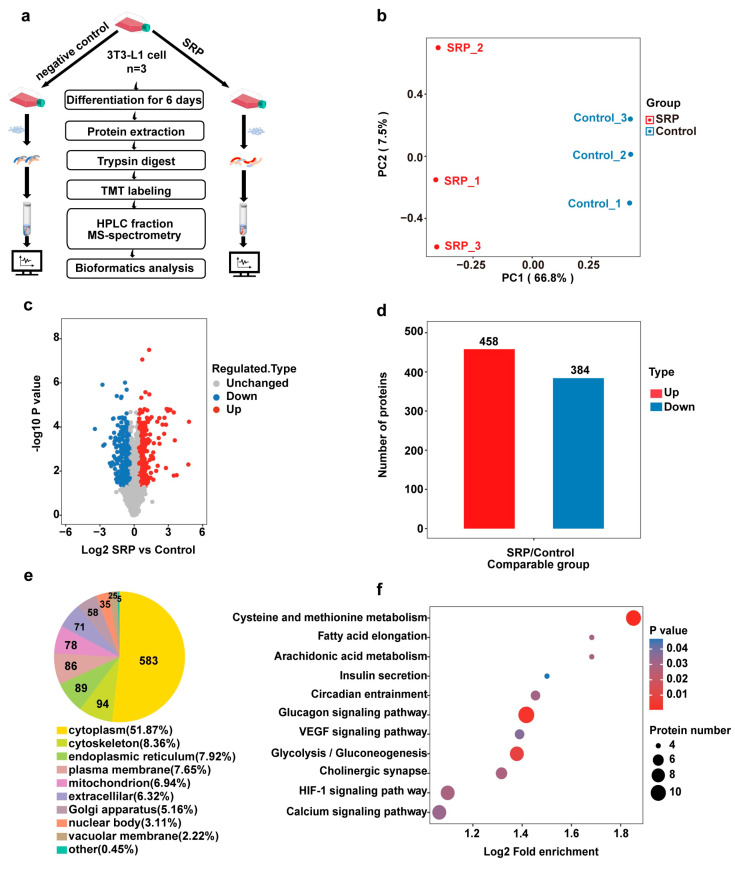
Identification of differentially expressed proteins. (**a**) Flow chart of proteomic sequencing of SRP and control 3T3-L1 cells. (**b**) Plot of the PCA (principal component analysis) distribution of all samples. (**c**) Quantitative volcano diagram of differentially expressed proteins. (**d**) Statistical diagram of the number of differentially expressed proteins. (**e**) Subcellular location of differentially expressed proteins. (**f**) KEGG pathway analysis of downregulated proteins.

To investigate the mechanism of SRP action on lipid accumulation in 3T3-L1 cells, we performed liquid chromatography–tandem mass spectrometry (LC–MS) for quantitative proteomics analysis (Figure 4a). The results of principal component analysis (PCA) showed good reproducibility between the two groups (Figure 4b). The proteins were filtered by fold change >1.3 and significance at *p* < 0.05, yielding 842 differentially expressed proteins, of which 458 were upregulated and 384 were downregulated (Figure 4c,d). To resolve the functions of these differentially expressed proteins, we performed subcellular localization analysis and found that most of the proteins were localized in the cytoplasm (Figure 4e). Kyoto Encyclopedia of Genes and Genomes (KEGG) pathway enrichment analysis found that the downregulated proteins mediate regulation of glucagon, VEGF, and glycolysis signaling (Figure 4f).

### 2.5. SRP Inhibits Lipogenic Differentiation of 3T3-L1 Cells by Targeting Src to Downregulate VEGFR2 Expression and Phosphorylation of PI3K/AKT

**Figure 5:**
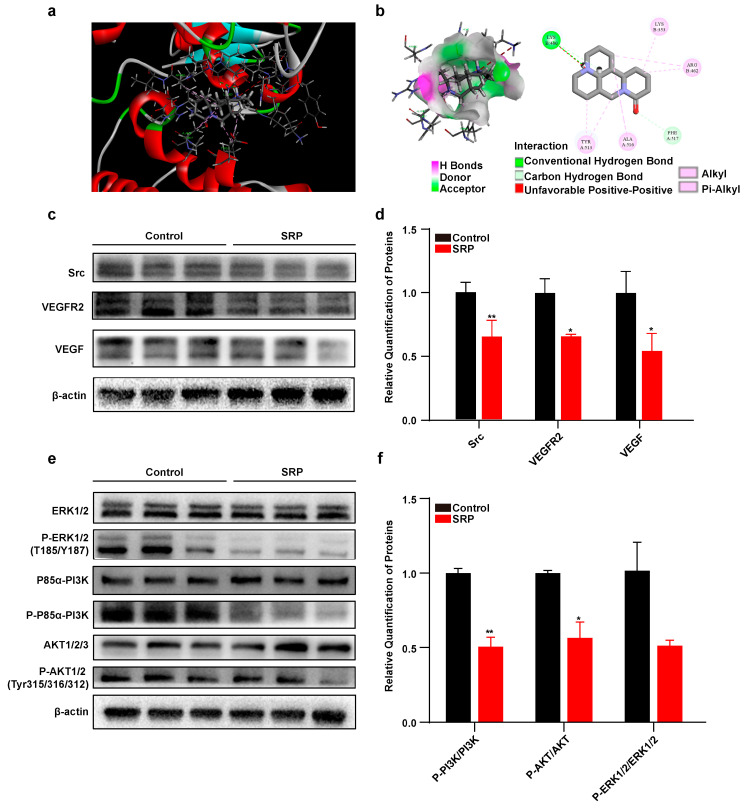
Sophoridine targets the Src protein domain and downregulates VEGFR2 expression and phosphorylation of PI3K/AKT. (**a**) Molecular docking diagram of sophoridine bound to the Src kinase domain (PDB: 1YOJ). Sophoridine stick model showing carbon (gray), hydrogen (white), oxygen (red), and nitrogen (blue) atoms. (**b**) Hydrogen bonds at receptor surfaces and differential interactions between sophoridine and the Src kinase domain. (**c**) Protein expression of Src, VEGFR2, and VEGF of 3T3-L1 cells. (**d**) Quantitative analysis of Western blot. (**e**) Protein expression of ERK1/2, P-ERK1/2 (T185/Y187), P85α-PI3K, P-P85α-PI3K, AKT1/2/3, P-AKT1/2 (Tyr315/316/312) of 3T3-L1 cells. (**f**) Quantitative analysis of Western blot. Data represent the mean ± SEM (*n* = 3). Statistical analysis was performed by Student’s *t*-test, * *p* < 0.05, and ** *p* < 0.01.

Differential protein function enrichment analysis led us to speculate that SRP might act through the VEGF signaling pathway. We examined the protein levels of VEGF and VEGFR and found that SRP significantly inhibited expression of VEGF and VEGFR2 (Figure 5c,d). *MAPK* [20], *Src* [21], and *Akt* [22] act as downstream effectors of *VEGF* signaling that also play important roles in fat deposition. Matrine, a tautomer of SRP, interacts with Src protein [23]. We predicted the molecular docking of SRP and Src kinase structural domains using Discovery Studio software (v19.1.0). Three-dimensional (3D) molecular dynamics simulations showed that SRP is embedded in the cleft of the Src kinase structural domain (Figure 5a). Alkyl and Pi-Alkyl interactions also formed between residues of SRP and the Src kinase domain, including Lys353, Tyr462, Ala513, and Tyr516. SRP also formed carbon-hydrogen bond interactions with Src Phe517 and Lys460. The B-chain Lys460 residue also exhibited hydrogen-bonding interactions with SRP (Figure 5b). We also found that SRP significantly inhibited protein expression of Src (Figure 5c,d). Finally, we examined downstream phosphorylation levels and found that SRP significantly downregulated the ratios of P-AKT/AKT and P-PI3K/PI3K. These results revealed that SRP inhibits lipogenic differentiation of 3T3-L1 cells by downregulating VEGFR2 expression and PI3K/AKT phosphorylation via Src protein binding.

## 3. Discussion

In recent years, major changes have taken place in the structure of the human diet. Being overweight and obese have become major problems worldwide, carrying additional risks of cardiovascular and cerebrovascular diseases, type 2 diabetes, non-alcoholic fatty liver, and other chronic diseases [24]. Therefore, it is very important to find small molecule drugs that can effectively control obesity and improve metabolic disorders. Excess dietary fat cannot be converted into other forms of macronutrients or excreted from the body; hence, it must either be stored or oxidized. Healthy mammals store surplus energy in the form of triacylglycerol (TAG) within lipid droplets located in adipocytes, rather than undergoing oxidation, ultimately resulting in weight gain. In this study, we explored the effects of SRP on bodyweight and metabolic homeostasis in mice with diet-induced obesity and report the underlying mechanism by which SRP inhibits adipocyte differentiation.

SRP is a tetracyclo-quinolizidine alkaloid [25] that has been shown to resist steatosis. Similar alkaloids, such as matrine, have been extensively researched for their effects on lipid metabolism [13,26,27,28]. Matrine reduced epididymal fat and lowered hyperglycemia in a mouse model of T2D induced by HF-STZ [26]. This is consistent with our findings that SRP reduces adiposity and ameliorates metabolic disturbances in mice with diet-induced obesity (DIO). However, some studies have shown that low doses of SRP can affect the weight and appetite of mice. When the dose exceeds 50 mg/kg, mice have reduced spontaneous activities, close their eyes, curl up, and even die [29,30]. Interestingly, we found a significant increase in brown fat weight in SRP-treated DIO mice, perhaps by promoting fat thermogenesis similar to several other natural molecules with anti-obesity effects, celastrol [31,32], berberine [33,34], matrine [13], and ginsenoside [35,36].

Additionally, 3T3-L1 cells have been used extensively in studies of the effects of compounds or nutrients on adipogenesis [37]. In this study, 3T3-L1 cells were used to simulate the effect of SRP on adipocyte proliferation and differentiation. Many studies have shown that SRP can inhibit the proliferation of gastric [12], pancreatic [9], and ovarian cancer cells [38]. We also found that SRP inhibits the proliferation of 3T3-L1 cells by reducing the number of cells in S phase and inhibiting the expression of proliferation-related genes. Matrine inhibits 3T3-L1 preadipocyte differentiation by inhibiting ERK1/2 phosphorylation and downregulating PPARγ and C/EBPα expression, resulting in reduced lipid accumulation in 3T3-L1 cells [39]. Matrine also reduces the expression of key adipogenic enzymes SREBP1c, SCD1, and FAS in the liver and inhibits lipid synthesis, thus inhibiting lipid accumulation in the liver [26]. However, there are no reports on the role of SRP in fat deposition. We found that SRP inhibited the formation of lipid droplets in 3T3-L1 cells and suppressed the expression of lipogenic marker genes.

To investigate the mechanism by which SRP inhibits adipocyte differentiation, we performed proteomic sequencing of 3T3-L1 cells treated with SRP during differentiation. KEGG enrichment analysis revealed that these differentially downregulated proteins could be involved in biological processes such as glucagon signaling, VEGF signaling, and glycolysis. The *VEGF/VEGFR2* pathway activates thermogenic programs in adipose tissue, thereby protecting mice against obesity [40,41]. In contrast, the *VEGF/VEGFR2* signaling axis is inhibited in diabetes-related complications. ROS generated by hyperglycemic states promote the activation and subsequent degradation of VEGFR2, and ROS-induced activation of *VEGFR2* signaling is mediated by Src family kinases [42]. Protein levels of Src were significantly increased in HFD-fed mice [43], and in human preadipocytes, inhibition of Src expression reduced the expression of adipogenic genes in early and late differentiation [44]. Thus, Src may be critical in regulating adipocyte generation. In this study, we found that SRP significantly inhibited expression of Src, VEGF and VEGFR2, which is consistent with previous findings. It has been shown that matrine can interact with the Src kinase structural domain to inhibit Src activity and downregulate MAPK/ERK, JAK2/STAT3, and PI3K/AKT phosphorylation signaling pathways, thereby inhibiting the proliferation of cancer cells [23]. We predicted the interaction between SRP and Src and found strong hydrogen bonding between the Lys460 residue of the Src B chain and sophoridine. We also found that SRP inhibited phosphorylation of PI3K/AKT. However, our study also has certain limitations. The concentrations we used on the 3T3-L1 cell line were not related to the concentrations used in mouse gavage experiments. We only used healthy controls on the 3T3-L1 cell line, but this does not indicate that the concentrations used on the cell line are also applicable in vivo.

## 4. Materials and Methods

### 4.1. Ethic Statement

All animal experiments were approved by the Institutional Animal Care and Use Committee of the Northwest A&F University (approval number: NWAFU-314021167). We had complied with all relevant ethical regulations for animal experimentation.

### 4.2. Animal Experiments and Methods

Six-week-old male C57BL/6J mice were purchased from the Laboratory Animal Center of Xi’an Jiaotong University. After 2 weeks of acclimatization, the mice were randomly allocated to a normal diet (ND) or HFD to induce obesity (HFD). After 12 weeks of feeding, the HFD mice were randomly allocated to subgroups that received 20 mg/kg saline (HFD) or SRP (HFD + SRP) by gavage daily for 11 weeks. Each group contained 6–10 mice and body weight and food intake were recorded weekly. At the end of the 11 weeks, all mice were anesthetized by ether inhalation and euthanized by cervical dislocation. Samples of the serum, brown adipose tissue (BAT), inguinal white adipose tissue (iWAT), epididymal WAT (eWAT), and liver tissue were collected immediately for future experiments. Throughout the experiment, standard temperature and humidity were maintained, and a 12 h light–dark cycle was applied.

### 4.3. Chemicals and Reagents

HFD (TP23400, 60% high-fat model) was ordered from Nantong Trophy Feed (Nantong, Jiangsu, China). Sophoridine (HPLC, ≥98.6%, CAS: 6882-68-4) was purchased from Chengdu Durst (Chengdu, China). Antibodies against peroxisome proliferator-activated receptor gamma (PPARγ, ab209350) and CCAAT enhancer binding protein alpha (C/EBPα, ab32358) were purchased from Abcam. Antibodies against adipose triglyceride lipase (ATGL, 2138) and glucose transporter 4 (GLUT4, 2213) were purchased from Cell Signaling (Danvers, MA, USA). Antibodies against fatty acid binding protein 4 (FABP4, sc-271529), cyclinB (sc-166152), P21 (sc-6246), cyclin-dependent kinase 4 (CDK4, sc-23896), cAMP-response element binding protein (sc-377154), and β-actin (sc-47778) were obtained from Santa Cruz (Dallas, TX, USA). Antibodies against CCAAT enhancer binding protein beta (CEBPβ, CY5065), cyclinD1 (CY5404), cyclinE2 (CY5821), c-Src (CY5459), vascular endothelial growth factor (VEGF, CY10042), vascular endothelial growth factor receptor 2 (VEGFR2, CY5385), phosphoinositide-3 kinase P85 alpha (P85α-PI3K, CY5355), AKT1/2/3 (CY5561), ERK1/2 (CY5487), P-P85α-PI3K (CY6427), P-AKT1/2 (Tyr315/316/312, AY0423), and P-ERK1/2 (Tyr185/Y187, CY5044) were purchased from Abways (Shanghai, China).

### 4.4. Cell Culture

Dulbecco’s modified Eagle’s medium (90%, Gibco, Carlsbad, MA, USA) and 10% FBS (Gibco) were used to culture proliferating 3T3-L1 cells, which were counted and seeded into plates. Drug treatment was applied when the culture density reached ~40% confluence and cells were collected 24 h later. Induction medium (10% FBS, 0.5 mM IBMX, 1 μM DEX, and 5 μg/mL insulin) was used to induce overgrowth for 2 days, and another induction medium (10% FBS, and 5 μg/mL insulin) was used to induce cell differentiation for 6 days at 5% CO_2_ and 37 °C.

### 4.5. Glucose Tolerance Test (GTT) and Insulin Tolerance Test (ITT)

The mice underwent GTT and ITT 9 and 11 weeks after initiation of SRP treatment. Intraperitoneal GTT (IPGTT) was performed after 16 h fasting. Glucose (2 g/kg body weight) was administered intraperitoneally, and blood glucose was measured at 0, 15, 30, 60, 90, and 120 min. For the intraperitoneal ITT (IPITT), mice were fasted for 5 h, injected with insulin (0.75 U/kg body weight), and blood glucose was measured at 0, 15, 30, 60, 90, and 120 min.

### 4.6. Hematoxylin and Eosin (H/E) Staining

Tissues and cells were fixed with 4% paraformaldehyde for 12 h, then dehydrated and embedded in paraffin before sectioning (10 µm). Finally, H/E staining was performed, and samples observed and photographed under a microscope.

### 4.7. Oil Red O Staining

Oil red O storage solution was purchased from Sigma-Aldrich (St. Louis, MO, USA) and stock solution was prepared in a 3:2 ratio with double-distilled water. After filter-sterilizing (0.22-μm membrane), diluted oil red O was added to the fixed cells and incubated for 30 min. Samples were washed three times with PBS and observed by camera-equipped microscopy. Isopropyl alcohol was used to release the dye for quantitative analysis by measuring absorbance at 510 nm with a microplate reader (PerkinElmer, Boston, MA, USA).

### 4.8. Boron Dipyrromethene Fluorescent Dye Staining (Bodipy)

The differentiated cells were washed 3 times with PBS and fixed with 4% paraformaldehyde for 30 min. Bodipy was added and samples incubated for 30 min in the dark. After 3–5 min washes with PBS, DAPI was added and incubated for 10 min. Samples were observed by camera-equipped microscopy.

### 4.9. 5-Ethynyl-2deoxyuridine (EdU) Staining

The cells were counted, seeded in 96-well plates, and treated with SRP at 40% confluence. After 24 h, reagent A was added and incubated for 2 h. The cells were removed and fixed according to the EdU kit protocol (RiboBio, Guangzhou, China).

### 4.10. Cell Counting Kit-8 Assay

The cells were counted and seeded in 96-well plates. When the cultures reached 40% confluence, SRP was added (10, 20, 30, 40, 50, 60, 70, 80, and 90 μM) and the plates were incubated for 24 h at 37 °C and 5% CO_2_. CCK-8 reagent (10%, Solarbio, Beijing, China) was added to each well under dark conditions and incubated for 2 h before absorbance was measured at 450 nm with a microplate reader.

### 4.11. Flow Cytometry

The cells were counted, seeded in 6-well plates, and cultured to ~40% confluence. SRP was added and the plates incubated for 24 h, then washed three times with PBS and collected in a 10-mL centrifuge tube. Samples were tested in duplicate. Collected cells were fixed with 70% cold ethanol and stored overnight at 4 °C. Next, the cells were placed on ice and stained with 50 mg/mL DAPI for 30 min before flow cytometry (Becton Dickinson, Franklin Lakes, NJ, USA).

### 4.12. Real-Time Quantitative Polymerase Chain Reaction (RT-qPCR)

Total RNA from cell and tissue samples was purified using RNAiso Plus (TaKaRa, Otsu, Japan) and reverse-transcribed according to kit instructions (TaKaRa). Reactions were performed in a Step One Plus system (ABI, Allston, MA, USA) with SYBR Green (Vazyme, Nanjing, China). Relative gene expression was calculated using the 2−ΔΔCt method with β-actin as reference. Primers are listed in Table 1.

### 4.13. Western Blotting

Protein was extracted from tissues and cells with RIPA lysis buffer (Beyotime, Shanghai, China) and quantified with a bicinchoninic acid (BCA) protein kit (Thermo Fisher Scientific, Waltham, MA, USA). The extracted proteins (20 μg) were separated by SDS–PAGE and transferred to PVDF membranes (Millipore, Bedford, MA, USA), blocked with milk for 2 h, incubated with primary antibody overnight at 4 °C, and then incubated with second antibody for 2 h. The original band diagram of the Western blot is in Supplementary file S1.

### 4.14. Proteomics Sequencing Analysis

Proteomics sequencing services were provided by Lianchuan Biotechnology (Hangzhou, China). For this, 3T3-L1 cells were treated with NC and SRP. Protein extraction, protease digestion, TMT/iTRAQ labeling, HPLC separation, liquid-phase tandem mass spectrometry, database search, and bioinformatics analysis were performed on cells induced to differentiate for 8 days after treatment.

### 4.15. Molecular Docking

Molecular docking studies were performed using the Discovery Studio 2019 Client. The Src kinase domain (1YOJ) crystal structure was obtained from the Protein Data Bank (PDB), without ligands and water. SRP was treated with ligand preparation and the LibDock model to investigate the spatial binding pattern of SRP and the Src kinase domain.

### 4.16. Statistical Analysis

All results were analyzed using GraphPad Prism 8 software (GraphPad Software, San Diego, CA, USA) and data are expressed as means ± SEM. Statistical significance between multiple groups was analyzed by one-way ANOVA followed by Tukey’s post hoc tests. Statistical analyses for two groups were performed by Student’s *t*-test. *p* < 0.05 was considered statistically significant.

## 5. Conclusions

**Figure 6:**
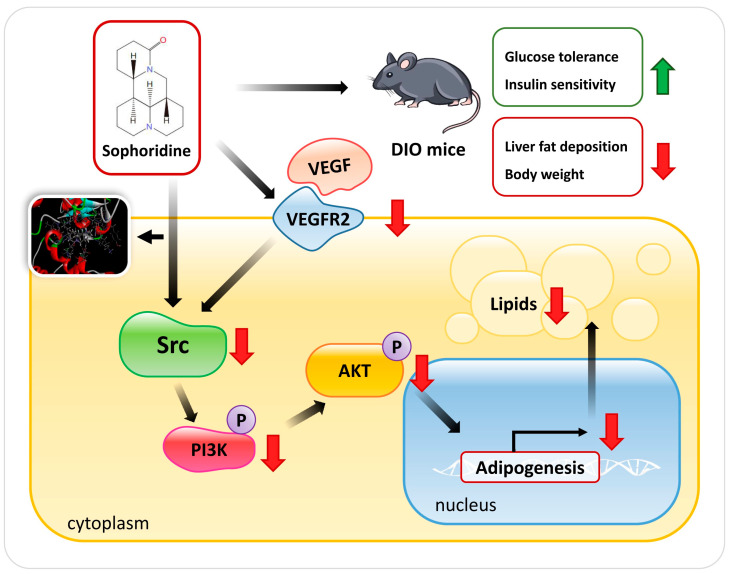
Sophoridine counteracts obesity via Src-mediated inhibition of VEGFR expression and PI3K/AKT phosphorylation.

In conclusion, this study shows that SRP inhibits VEGFR expression and PI3K/AKT phosphorylation by binding to the Src structural domain, thereby inhibiting adipocyte differentiation and somewhat ameliorating lipid accumulation in subcutaneous fat and the liver (Figure 6). This study provides evidence for a potential small molecule-based therapeutic approach for obesity.

## Figures and Tables

**Table 1:** Primer sequences used for gene expression analysis in this study.

Gene	Forward Primer (5′-3′)	Reverse Primer (5′-3′)
*SREBP1*	CGCTCTTGACCGACATCGA	GGCACGGACGGGTACATCTT
*PPARγ*	CCAAGAATACAAAGTGCGATCA	CCCACAGACTCGGCACTCAAT
*FASN*	AATGCAGACACCTTGGCACT	ACAGCTGTACTCTTGTTCTGGA
*C/EBPα*	TGGACAAGAACAGCAACGAG	TCACTGGTCAACTCCAGCAC
*C/EBPβ*	GAAGACGGTGGACAAGCTGA	TTGTGCTGCGTCTCCAGG
*FABP4*	AAGAAGTGGGAGTGGGCTTTG	CTCTTCACCTTCCTGTCGTCTG
*GLUT4*	GTGACTGGAACACTGGTCCTA	CCAGCCACGTTCATTGTAG
*ATGL*	TTCGCAATCTCTACCGCCTC	AAAGGGTTGGGTTGGTTCAG
*HSL*	GCTGGGCTGTCAAGCACTGT	GTAACTGGGTAGGCTGCCAT
*cyclinD*	TAGGCCCTCAGCCTCACTA	CCACCCCTGGGATAAAGCAC
*cyclinE*	GCTTGCTCCGGGGATGAAAT	GCGAGGACACCATAAGGAAATCTG
*cyclinB*	GCGTACCCTGACACCAATCTC	CTCCTCTTCGCACTTCTGCTC
*CDK4*	ATGGCTGCCACTCGATATGAA	TCCTCCATTAGGAACTCTCACAC
*P21*	AGTGTGCCGTTGTCTCTTCG	ACACCAGAGTGCAAGACAGC
*P27*	AGAAGCACTGCCGGGATATG	GACCCAATTAAAGGCACCGC

## Data Availability

Data from this study are included in the article.

## Supplementary Materials

The supporting information can be downloaded at: https://www.mdpi.com/article/10.3390/ijms25021206/s1.
