# Supplementary figures and images for: Sophoridine Counteracts Obesity via Src-Mediated Inhibition of VEGFR Expression and PI3K/AKT Phosphorylation
Source: Int J Mol Sci. 2024 Jan 19;25(2):1206. doi: 10.3390/ijms25021206 (PMC10816114; doi:10.3390/ijms25021206)

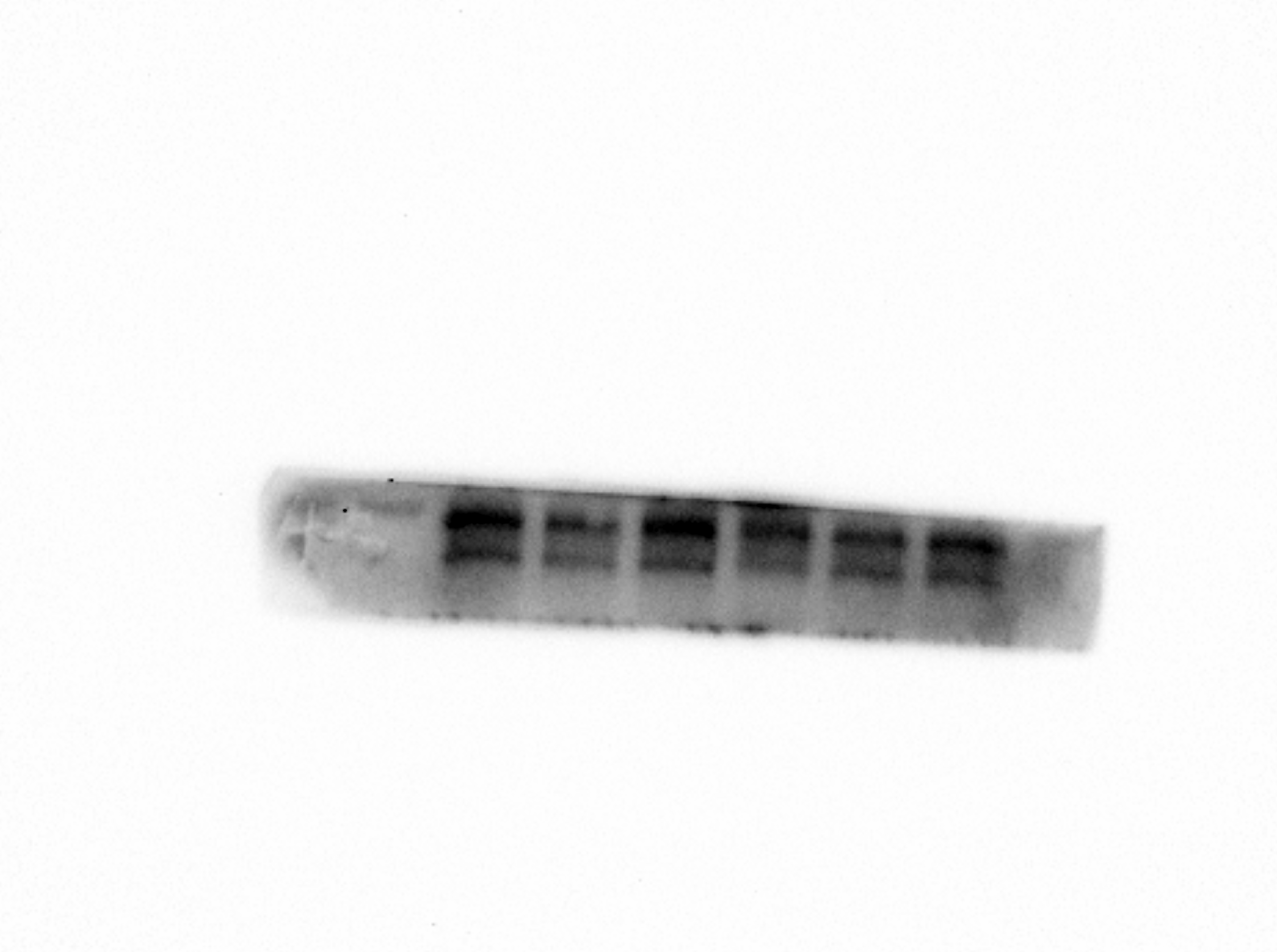

Supplement: Supplementary file 1 [file ijms-25-01206-s001.zip › Original Images for Blots/figure2e/CDK4.tif]

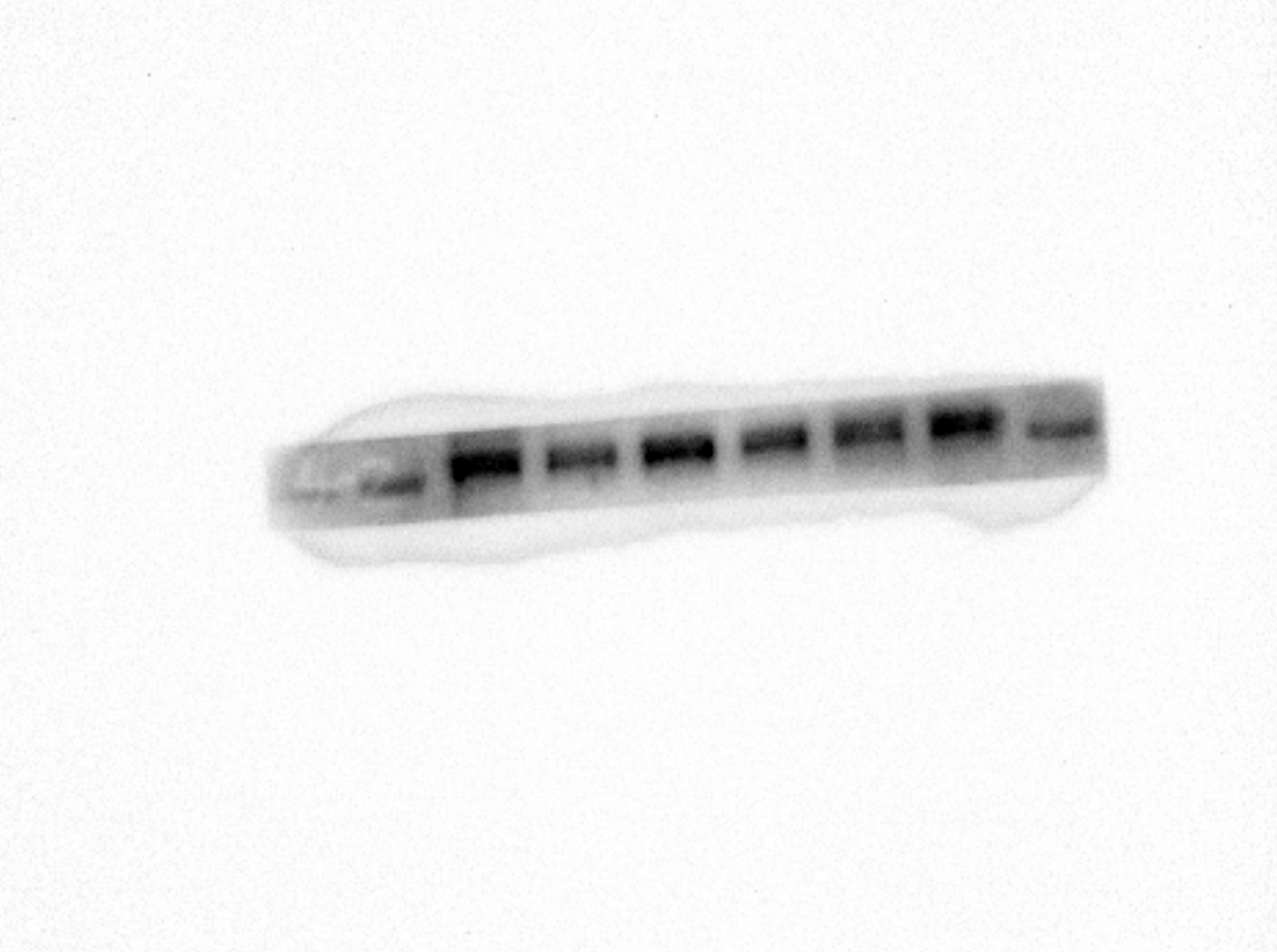

Supplement: Supplementary file 1 [file ijms-25-01206-s001.zip › Original Images for Blots/figure2e/CREB.tif]

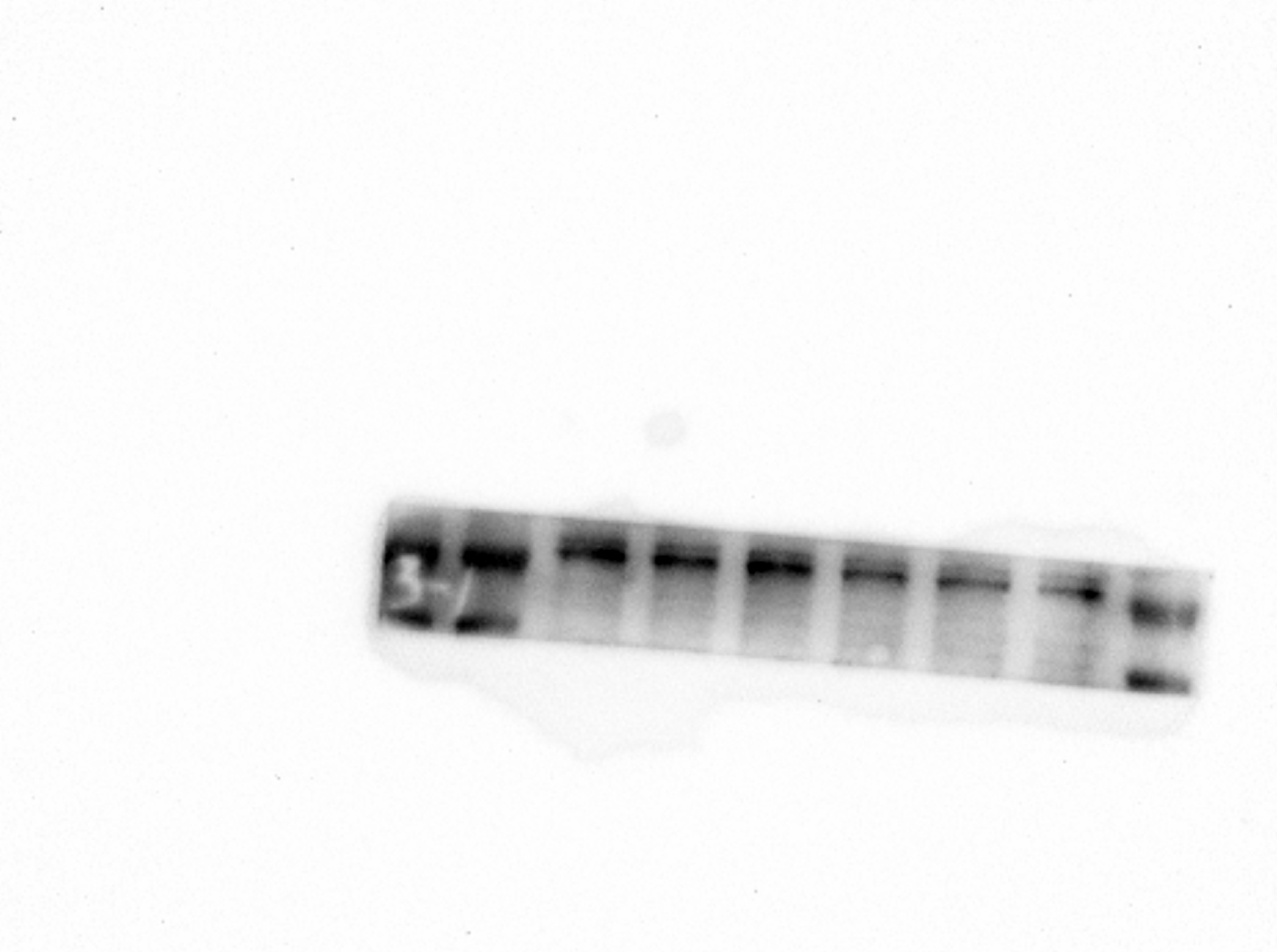

Supplement: Supplementary file 1 [file ijms-25-01206-s001.zip › Original Images for Blots/figure2e/CyclinB.tif]

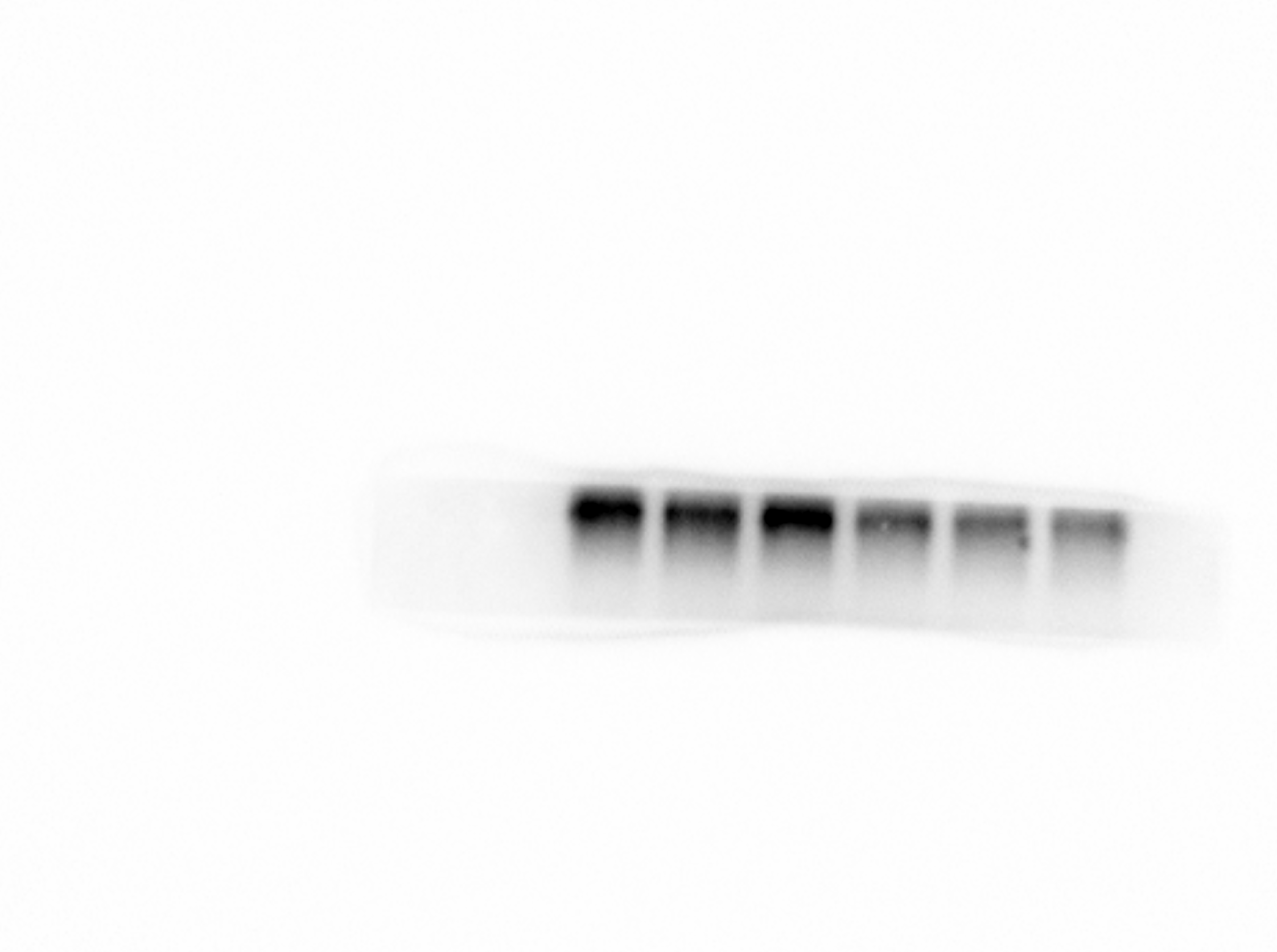

Supplement: Supplementary file 1 [file ijms-25-01206-s001.zip › Original Images for Blots/figure2e/CyclinD.tif]

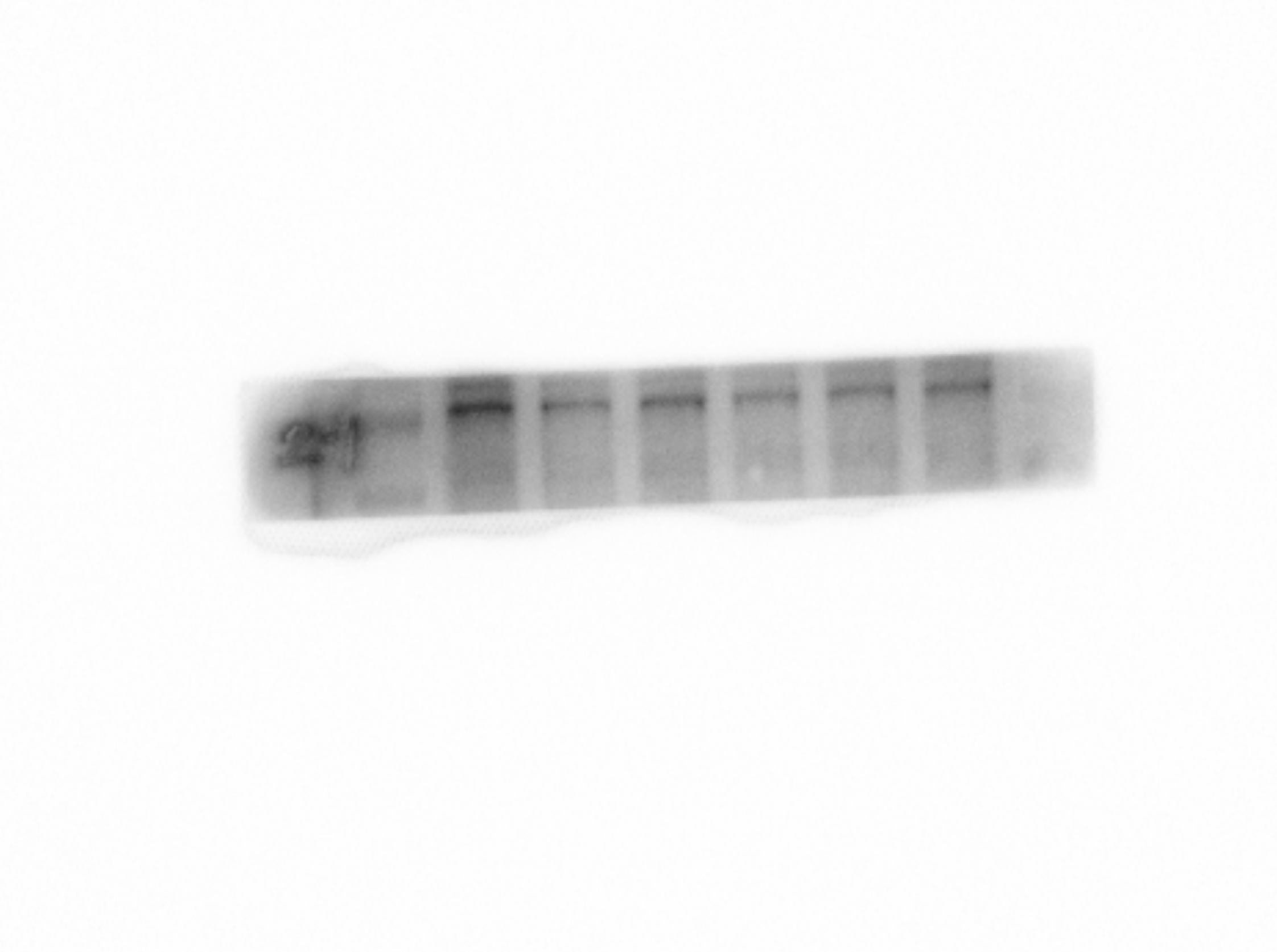

Supplement: Supplementary file 1 [file ijms-25-01206-s001.zip › Original Images for Blots/figure2e/CyclinE.tif]

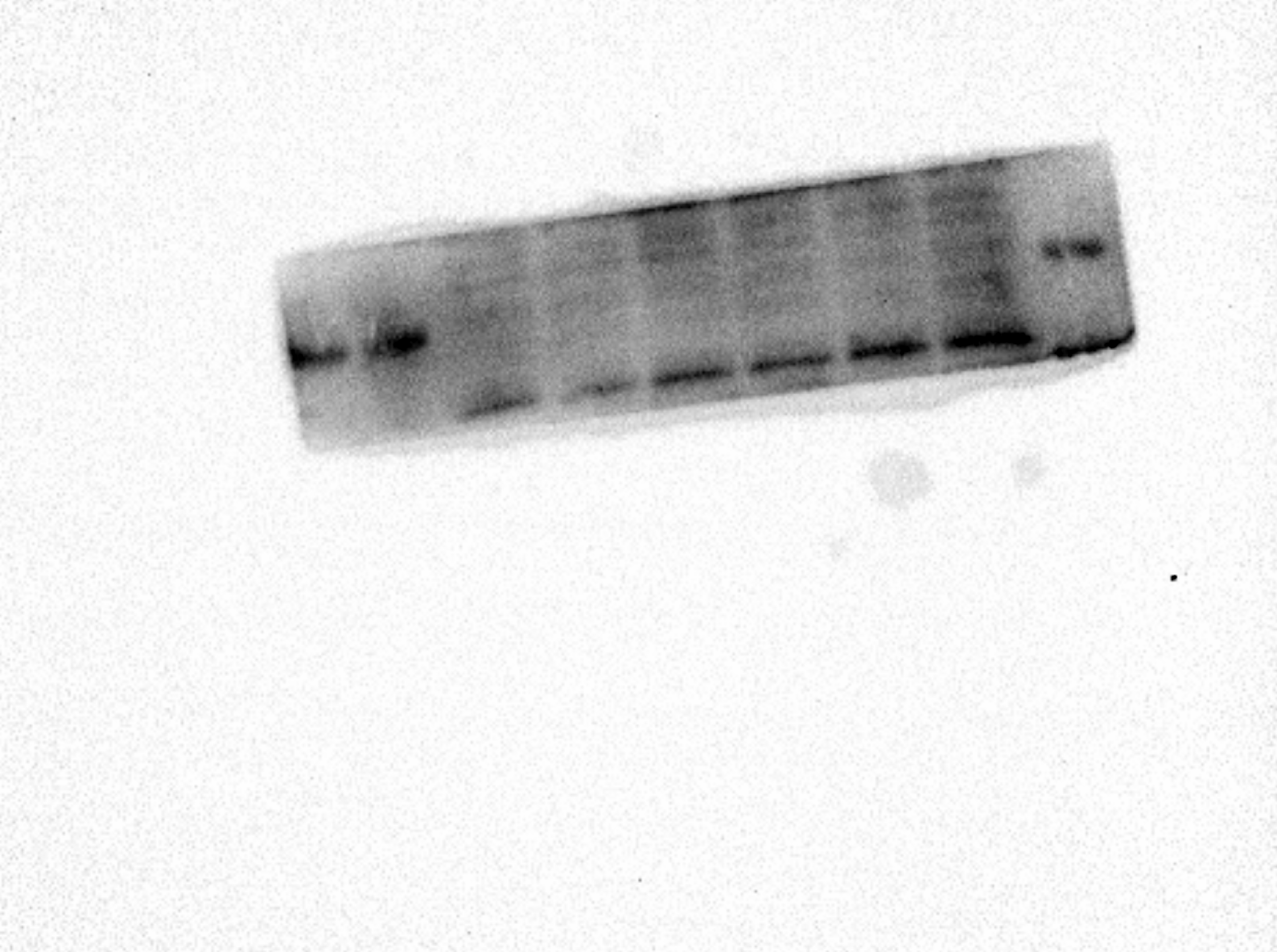

Supplement: Supplementary file 1 [file ijms-25-01206-s001.zip › Original Images for Blots/figure2e/P21.tif]

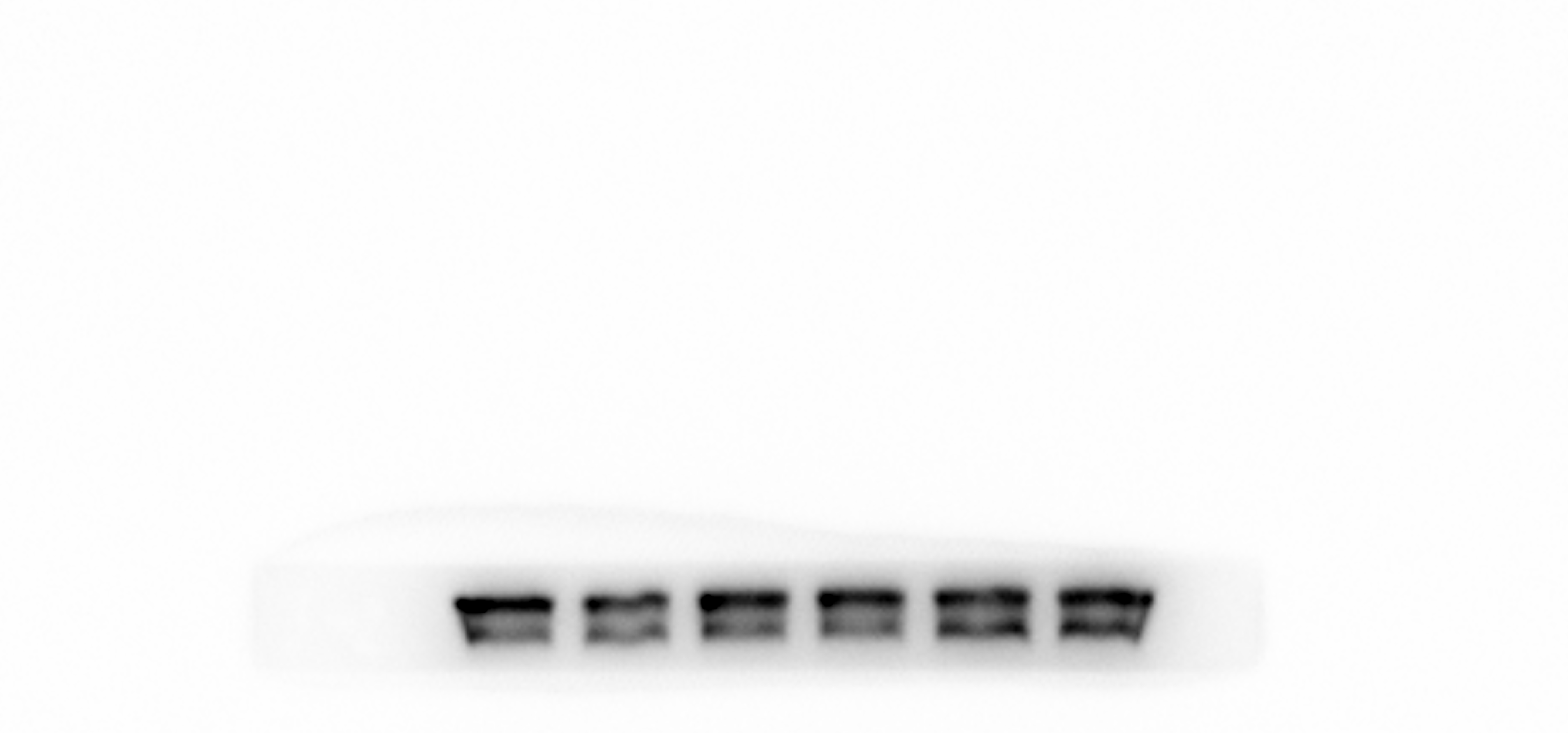

Supplement: Supplementary file 1 [file ijms-25-01206-s001.zip › Original Images for Blots/figure2e/β-actin.tif]

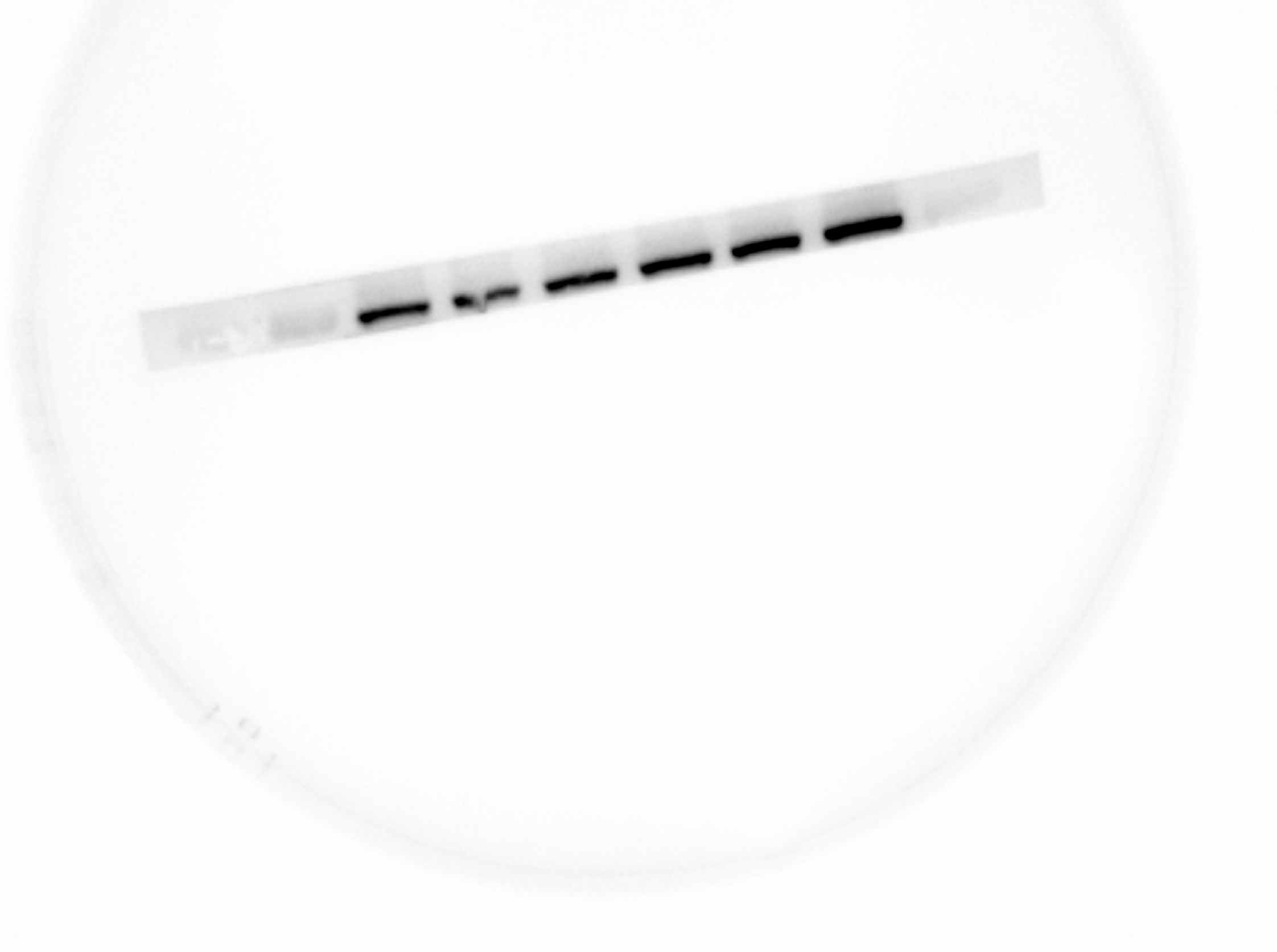

Supplement: Supplementary file 1 [file ijms-25-01206-s001.zip › Original Images for Blots/figure3a/ATGL.tif]

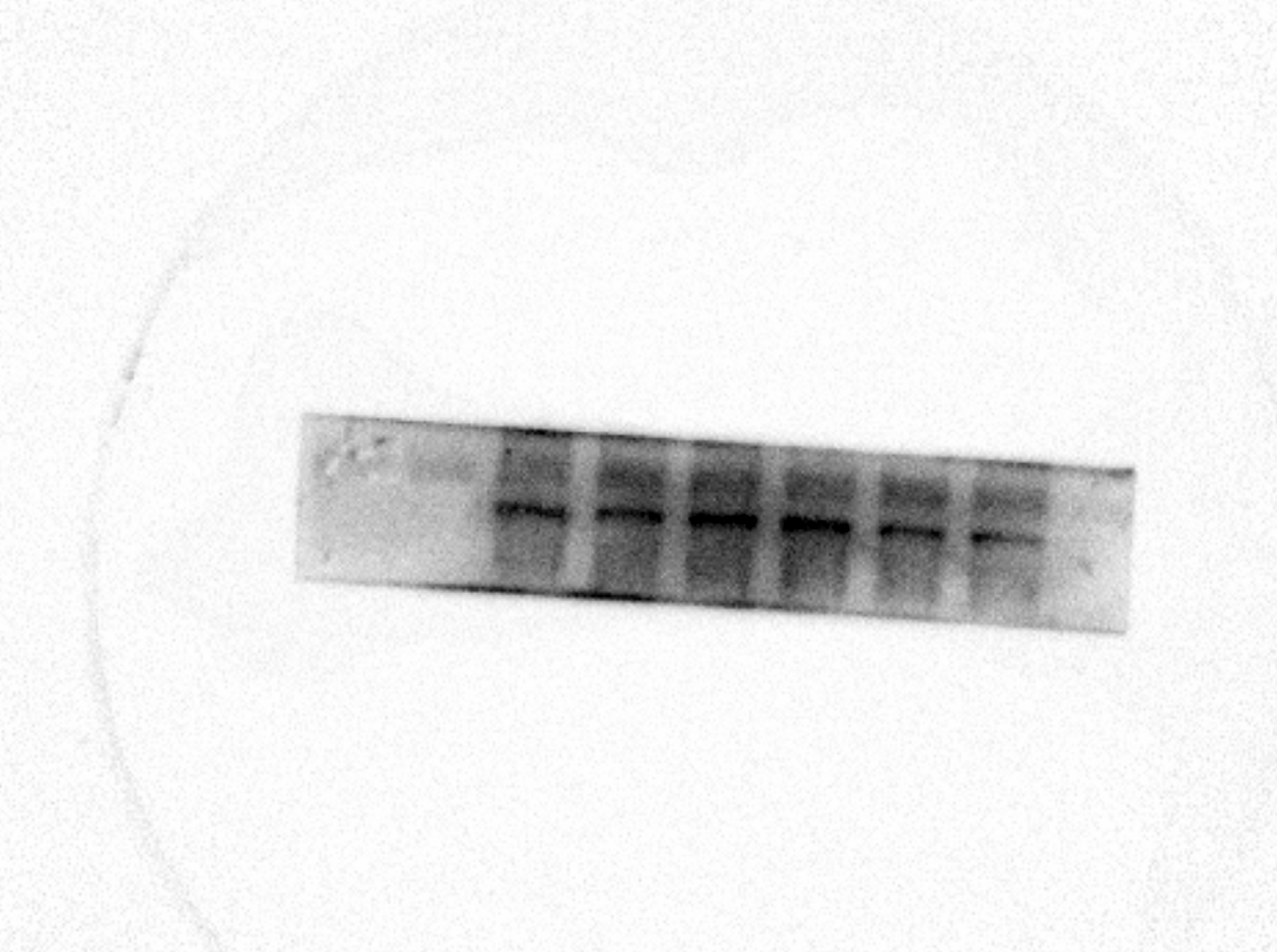

Supplement: Supplementary file 1 [file ijms-25-01206-s001.zip › Original Images for Blots/figure3a/CEBPα.tif]

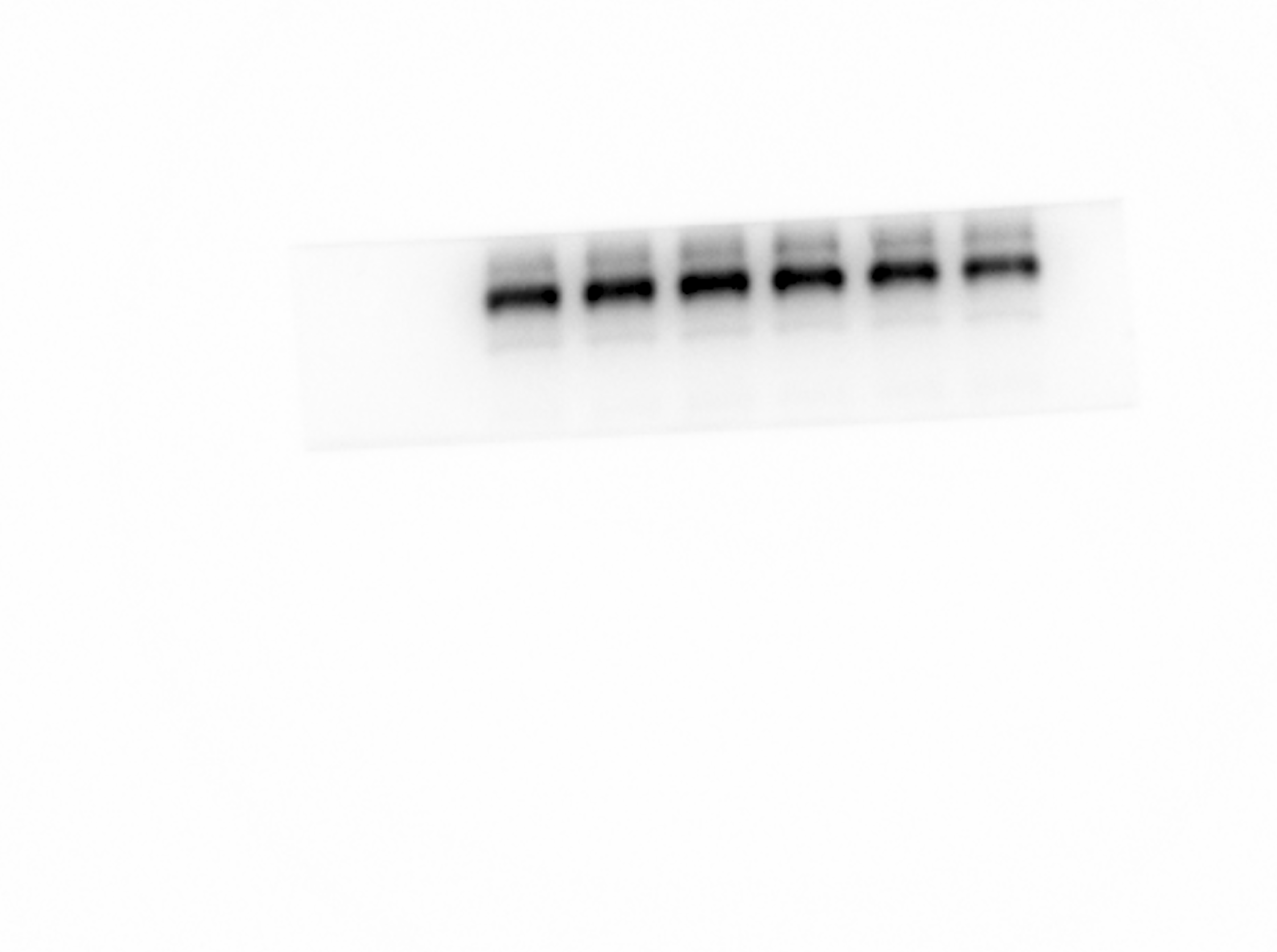

Supplement: Supplementary file 1 [file ijms-25-01206-s001.zip › Original Images for Blots/figure3a/CEBPβ.tif]

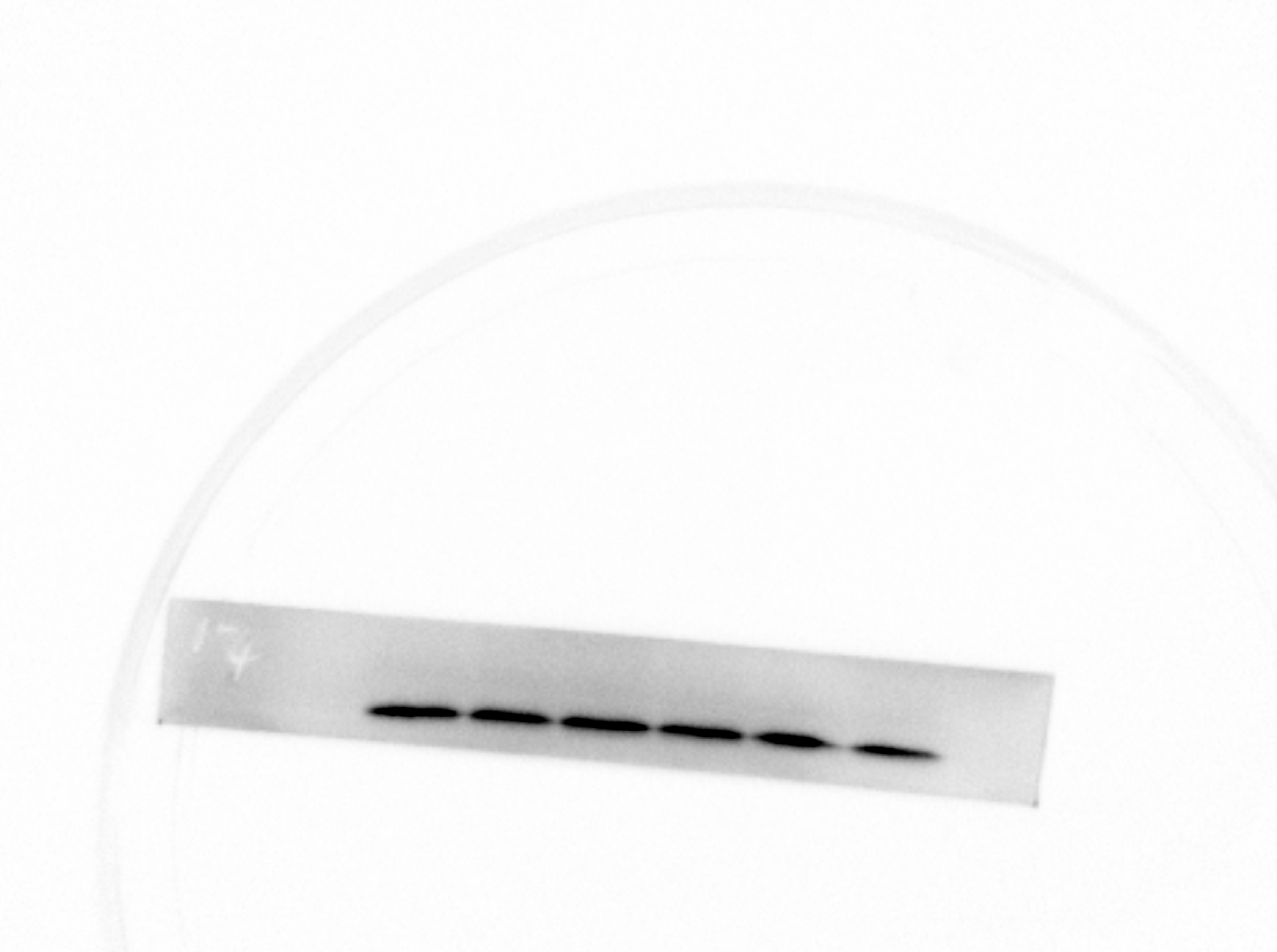

Supplement: Supplementary file 1 [file ijms-25-01206-s001.zip › Original Images for Blots/figure3a/FABP4.tif]

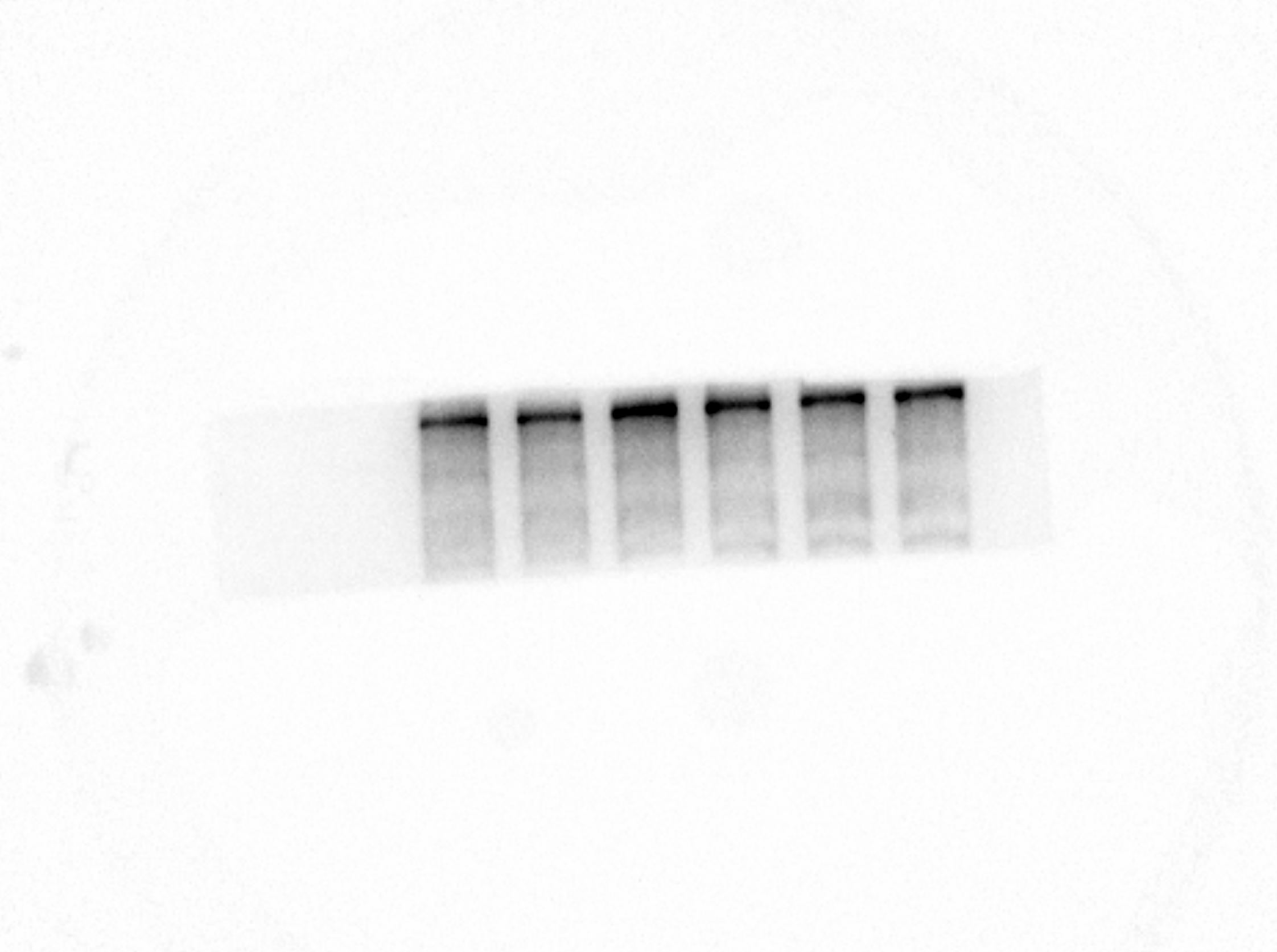

Supplement: Supplementary file 1 [file ijms-25-01206-s001.zip › Original Images for Blots/figure3a/FASN.tif]

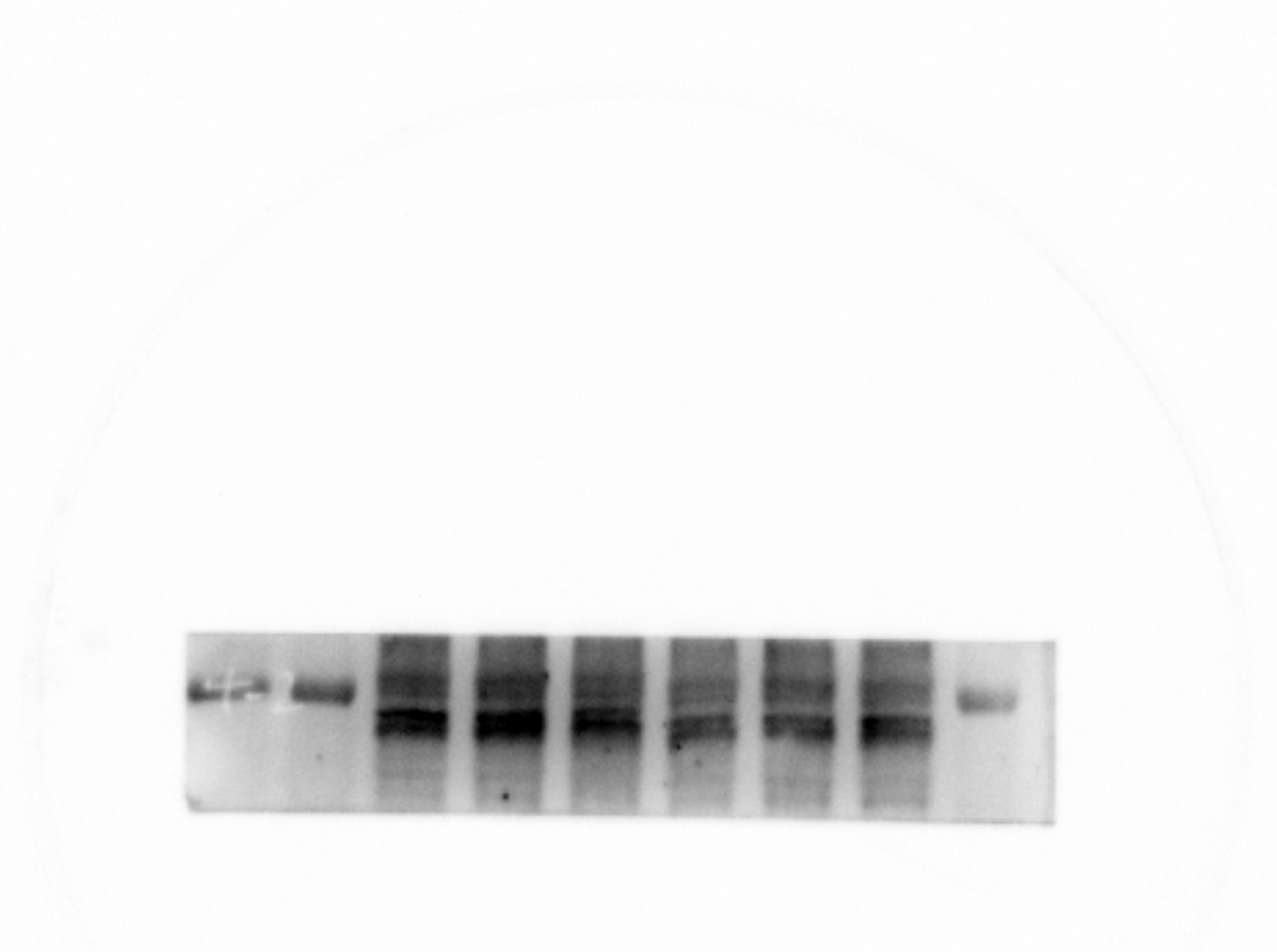

Supplement: Supplementary file 1 [file ijms-25-01206-s001.zip › Original Images for Blots/figure3a/GLUT4.tif]

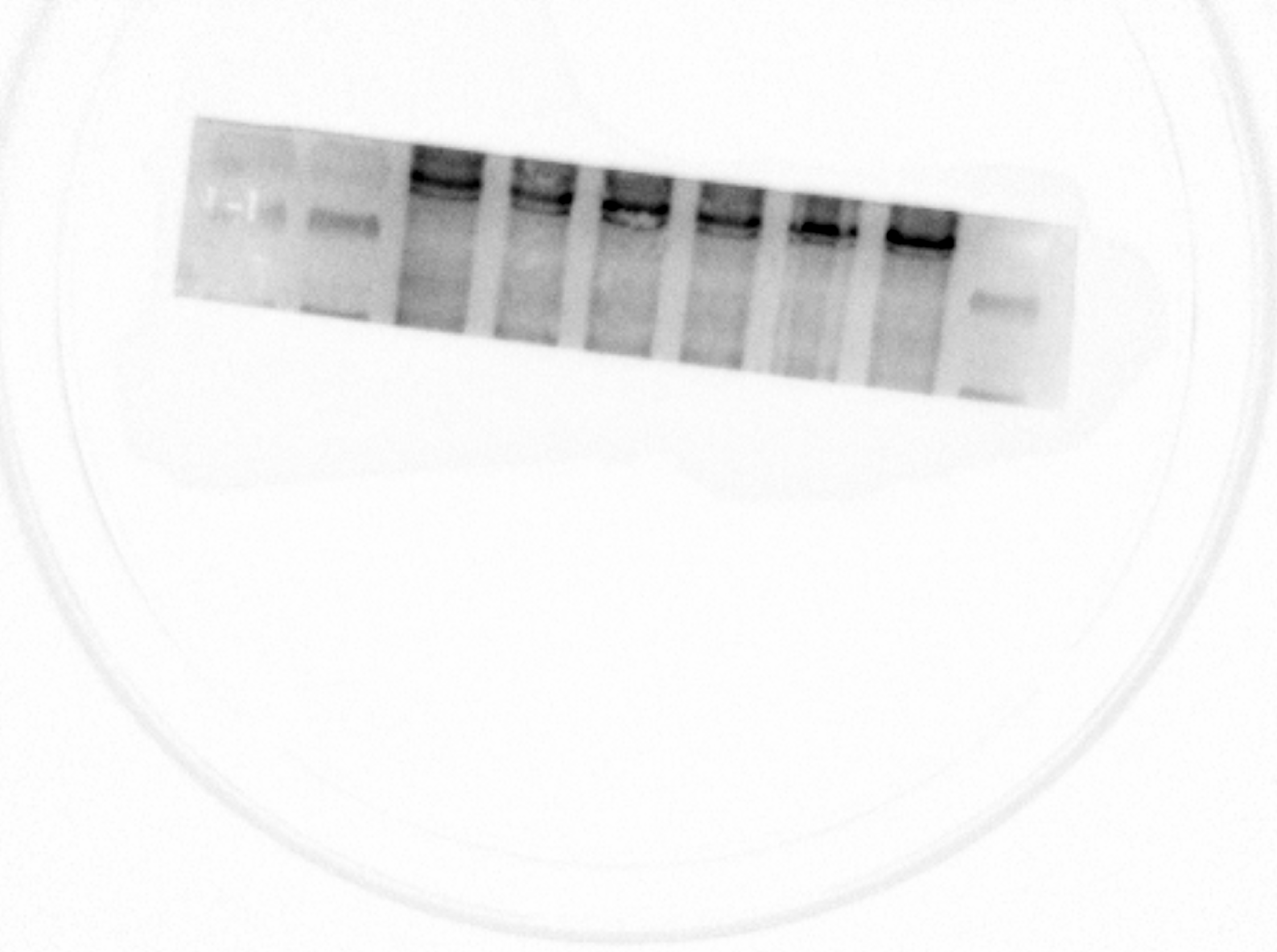

Supplement: Supplementary file 1 [file ijms-25-01206-s001.zip › Original Images for Blots/figure3a/HSL.tif]

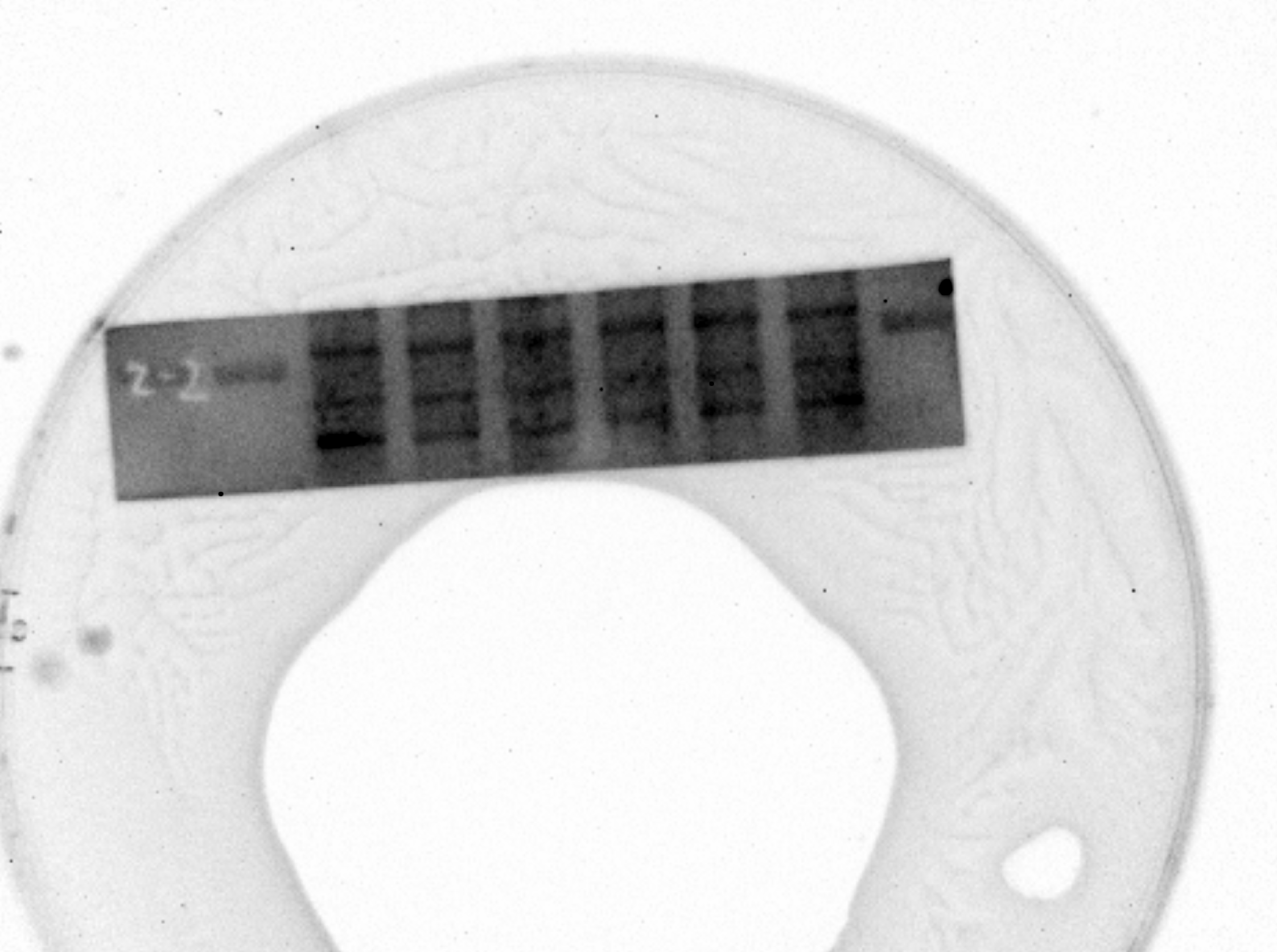

Supplement: Supplementary file 1 [file ijms-25-01206-s001.zip › Original Images for Blots/figure3a/PPARγ.tif]

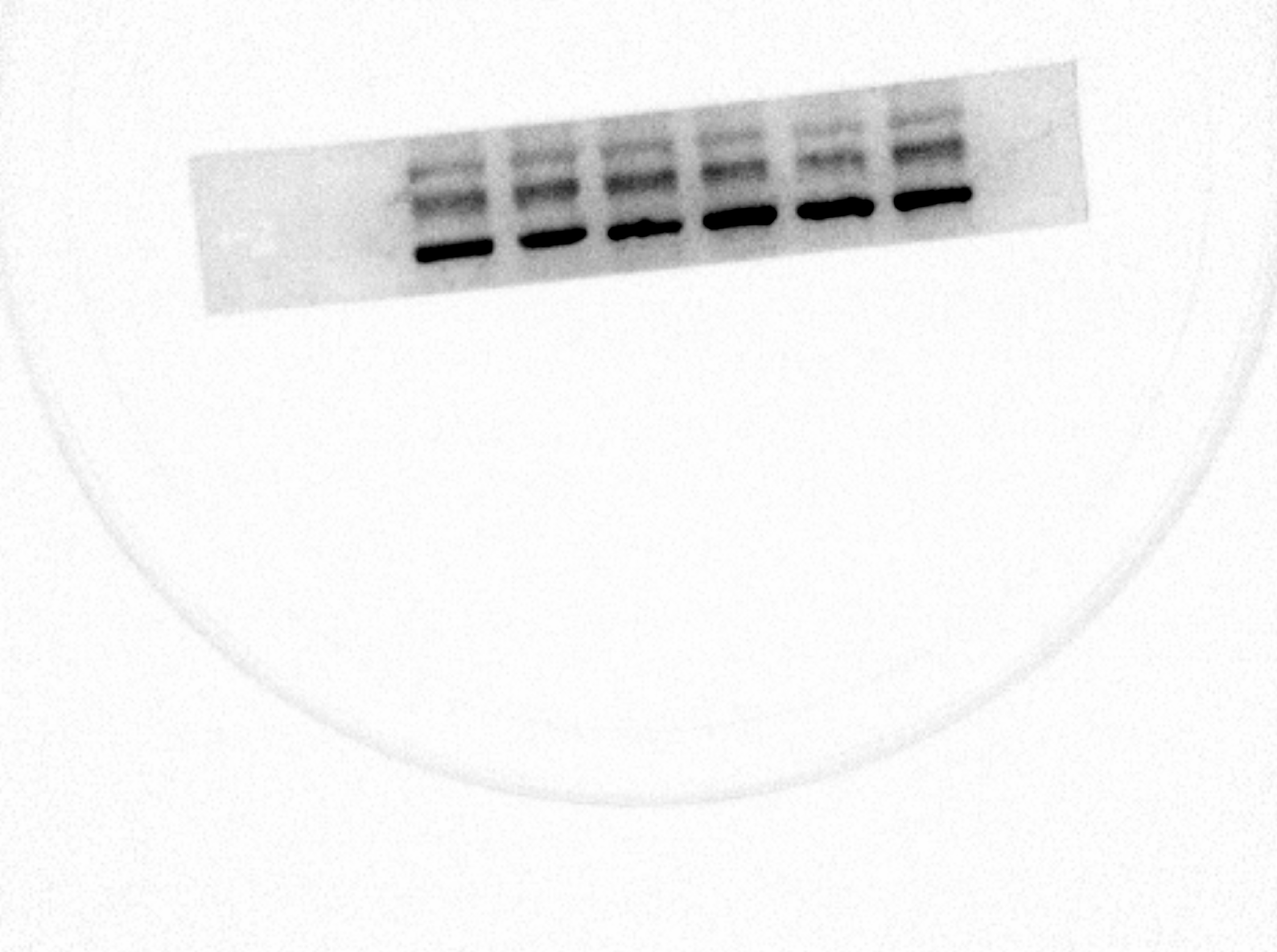

Supplement: Supplementary file 1 [file ijms-25-01206-s001.zip › Original Images for Blots/figure3a/β-actin.tif]

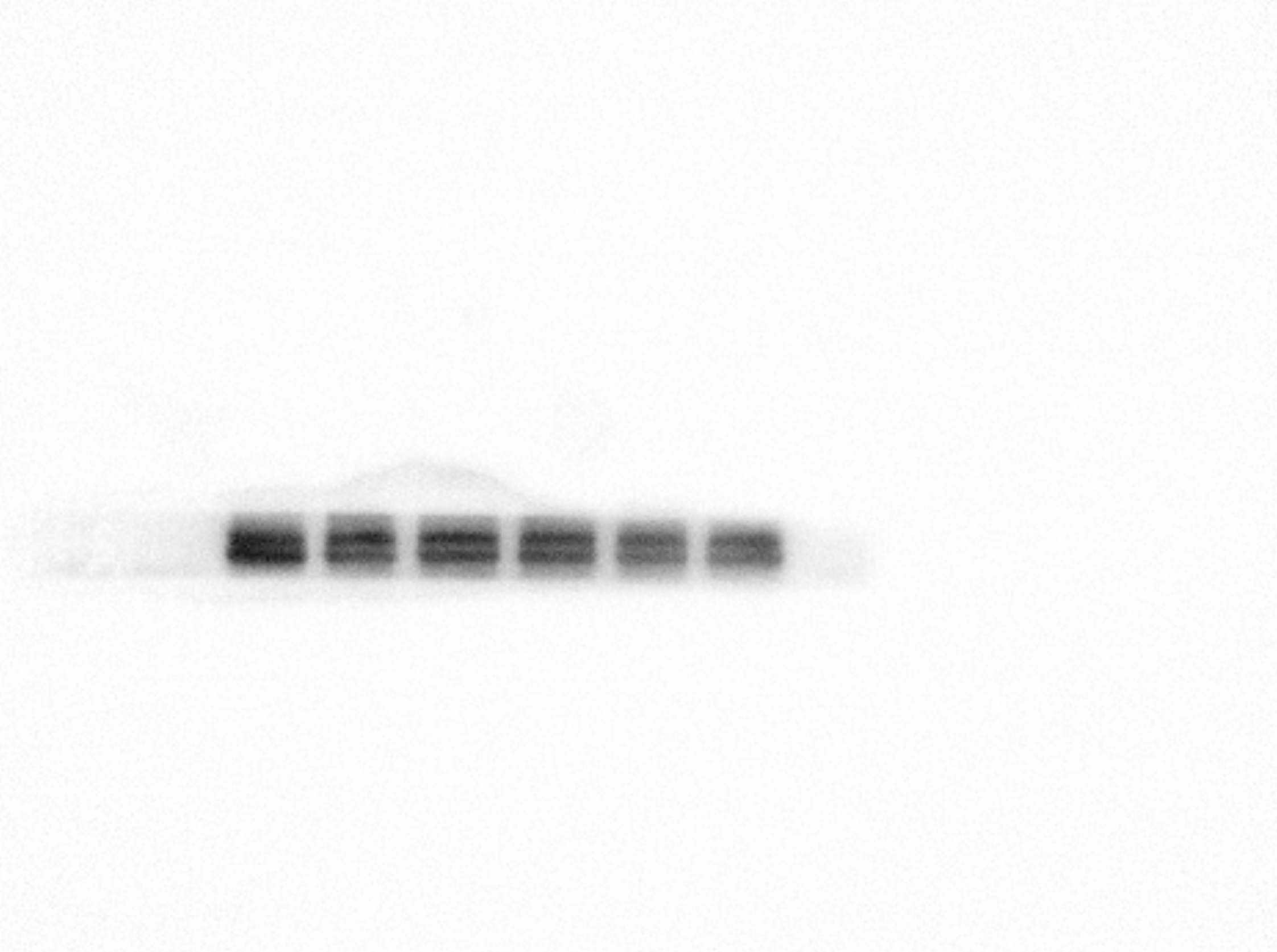

Supplement: Supplementary file 1 [file ijms-25-01206-s001.zip › Original Images for Blots/figure5c/Src.tif]

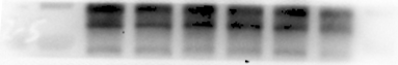

Supplement: Supplementary file 1 [file ijms-25-01206-s001.zip › Original Images for Blots/figure5c/VEGF.tif]

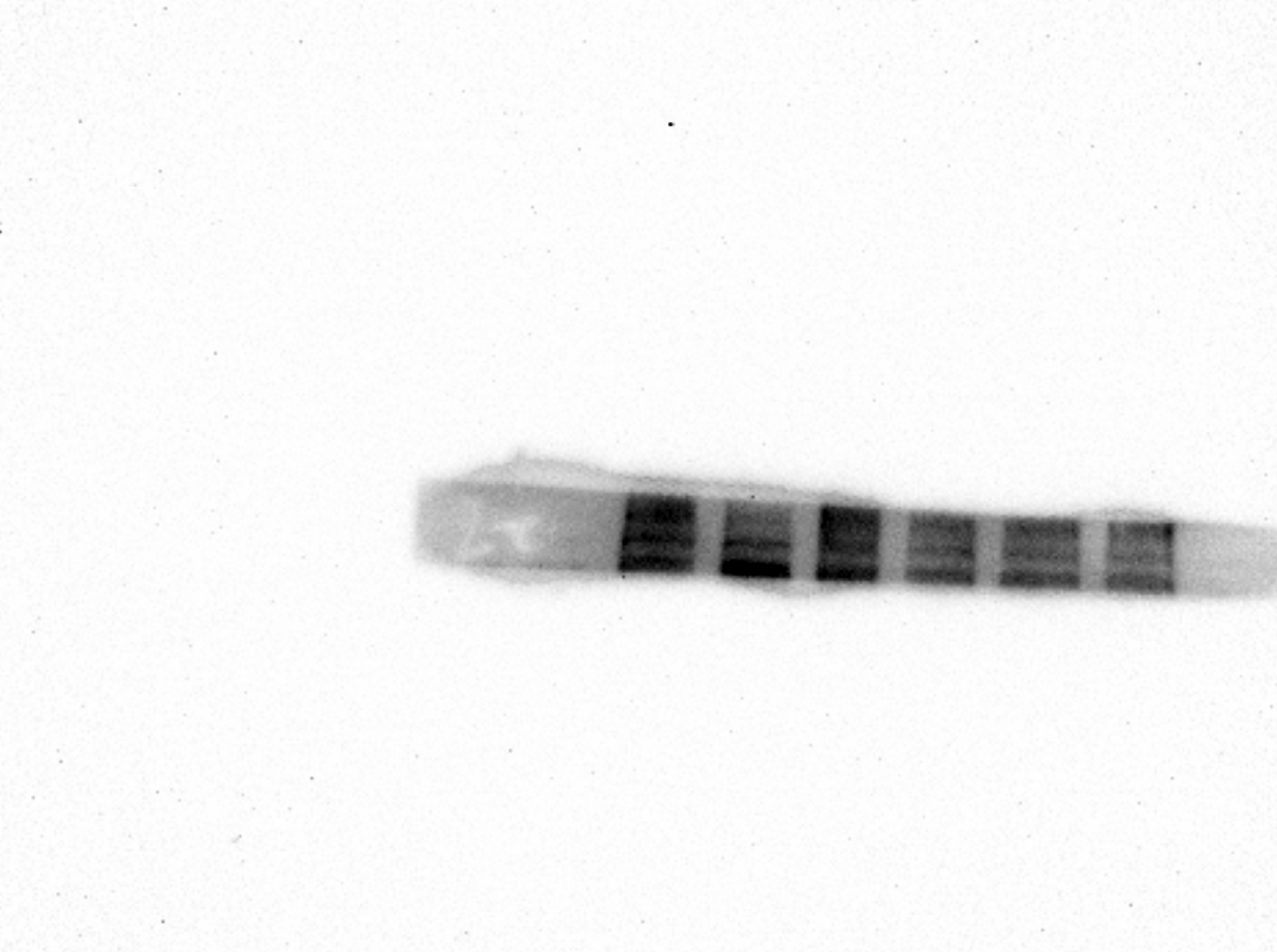

Supplement: Supplementary file 1 [file ijms-25-01206-s001.zip › Original Images for Blots/figure5c/VEGFR2.tif]

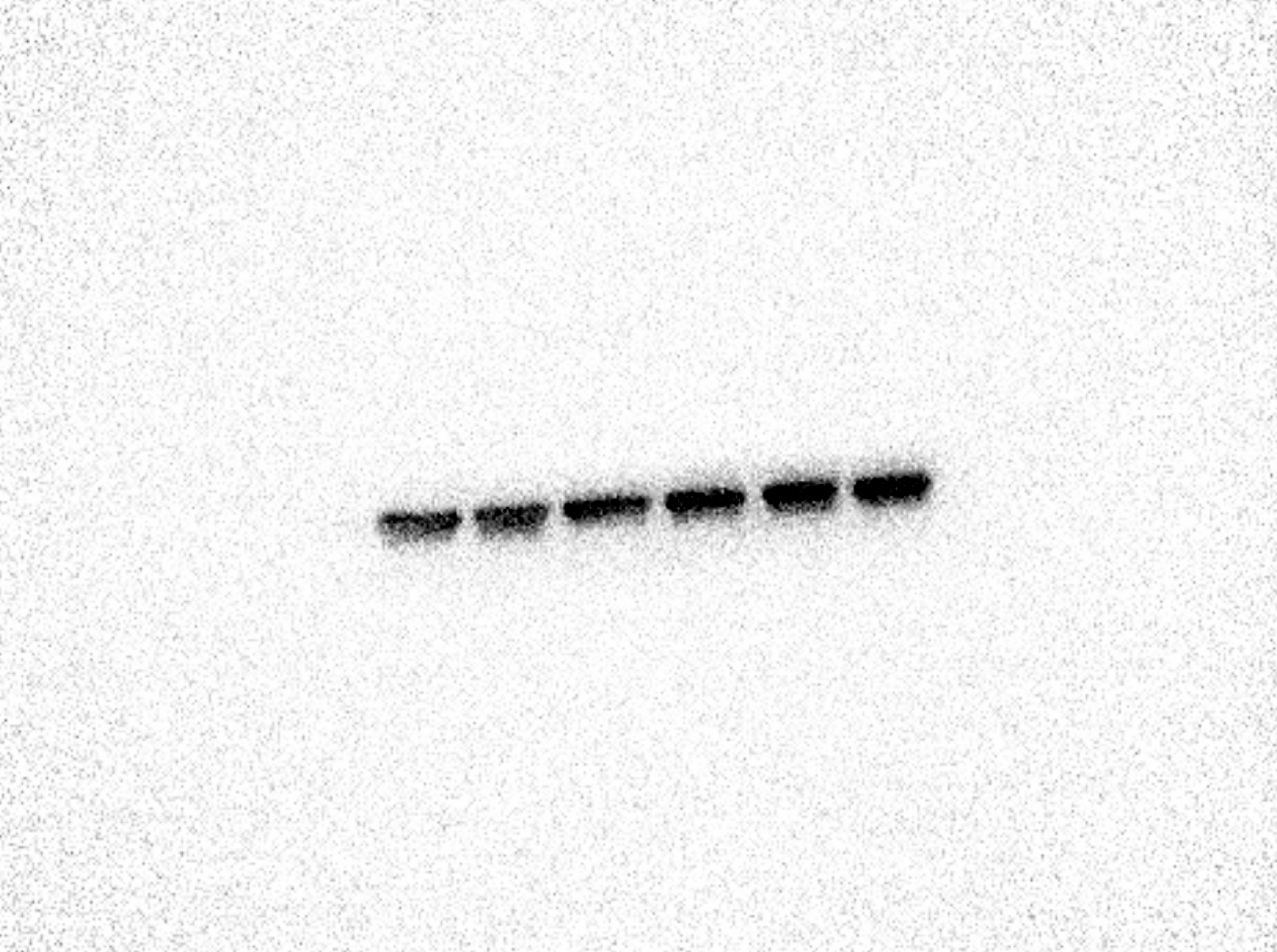

Supplement: Supplementary file 1 [file ijms-25-01206-s001.zip › Original Images for Blots/figure5c/β-actin.tif]

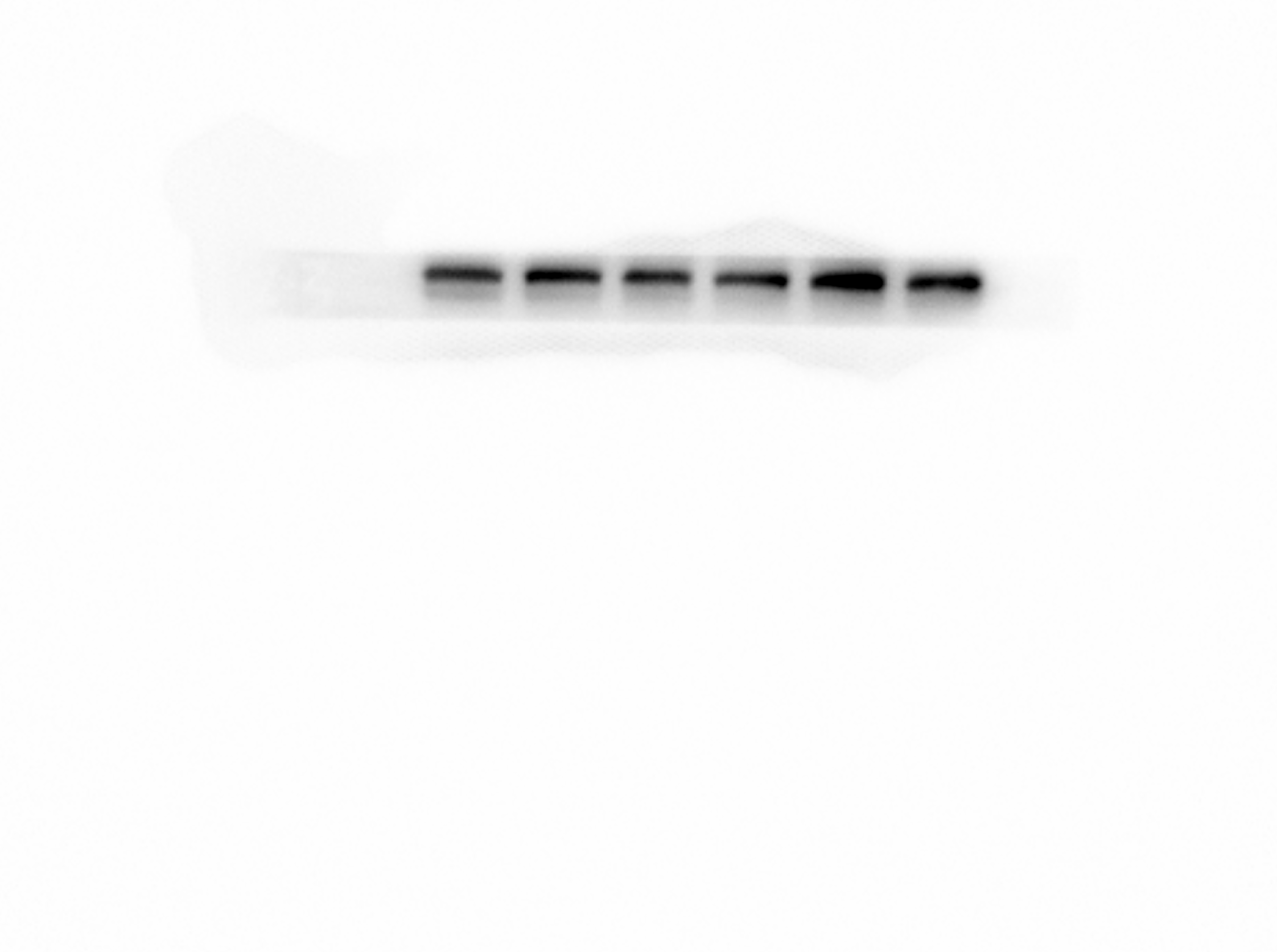

Supplement: Supplementary file 1 [file ijms-25-01206-s001.zip › Original Images for Blots/figure5e/AKT123.tif]

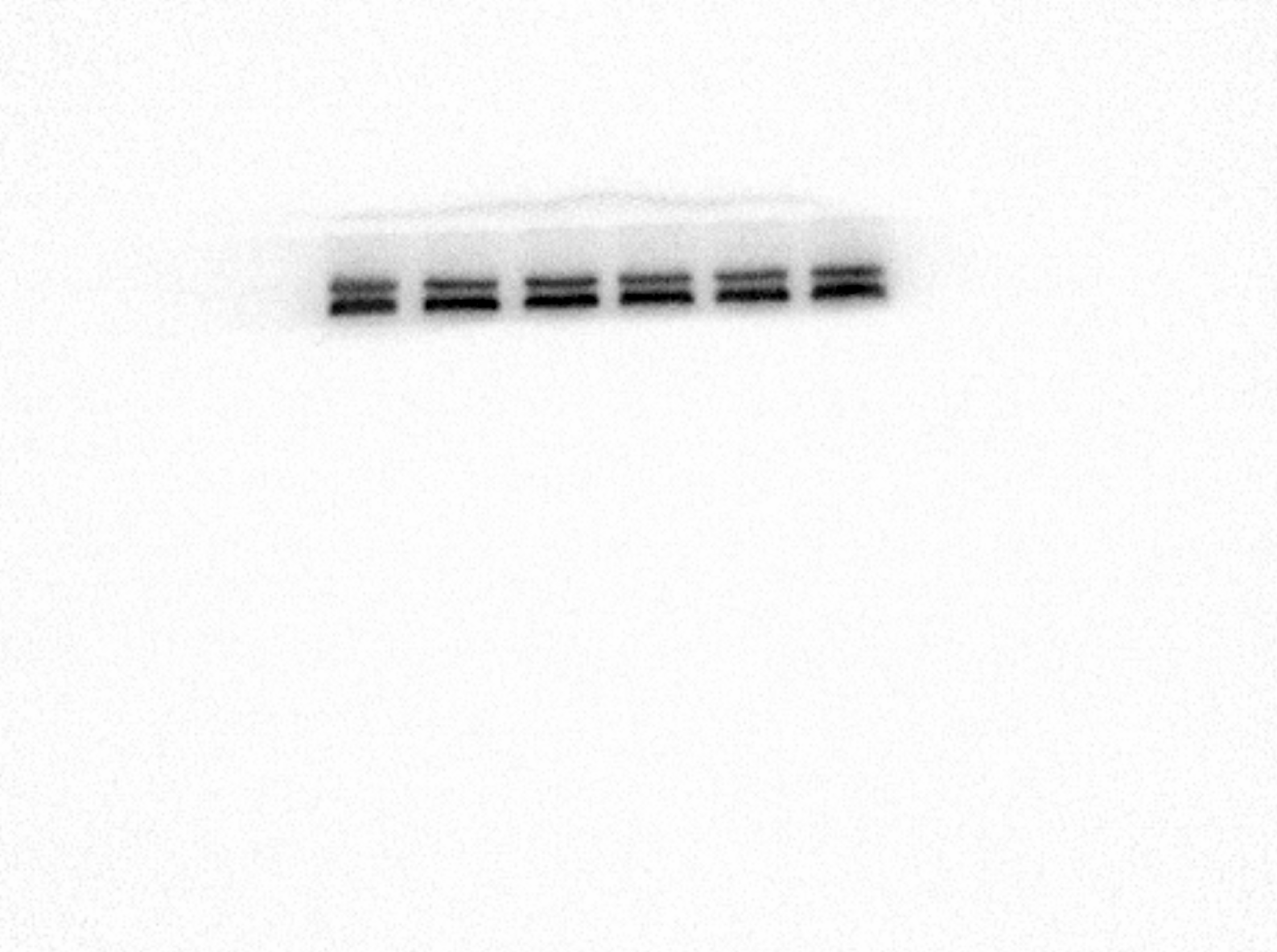

Supplement: Supplementary file 1 [file ijms-25-01206-s001.zip › Original Images for Blots/figure5e/ERK1,2.tif]

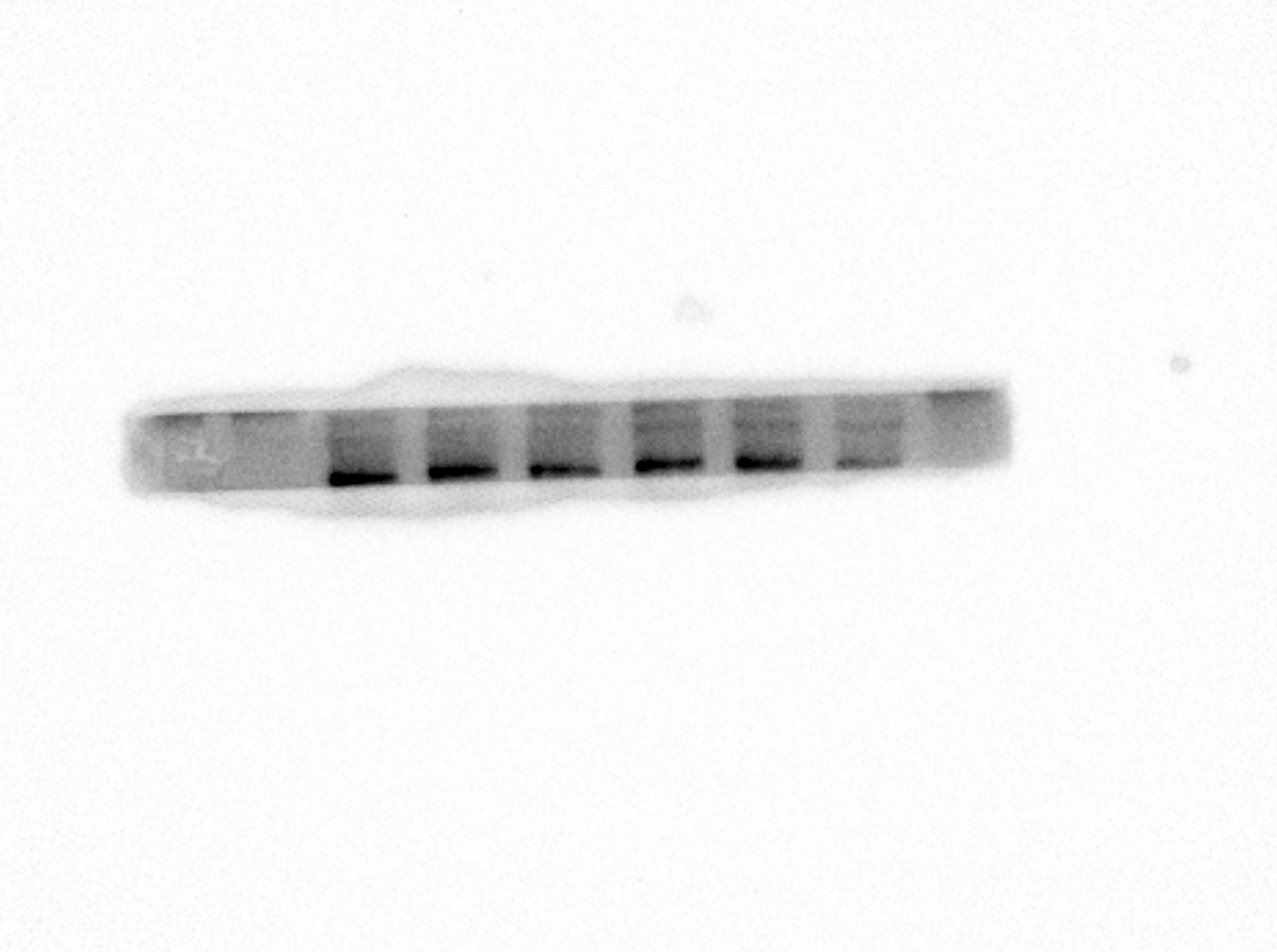

Supplement: Supplementary file 1 [file ijms-25-01206-s001.zip › Original Images for Blots/figure5e/P-AKT1,2(Tyr315,316,312).tif]

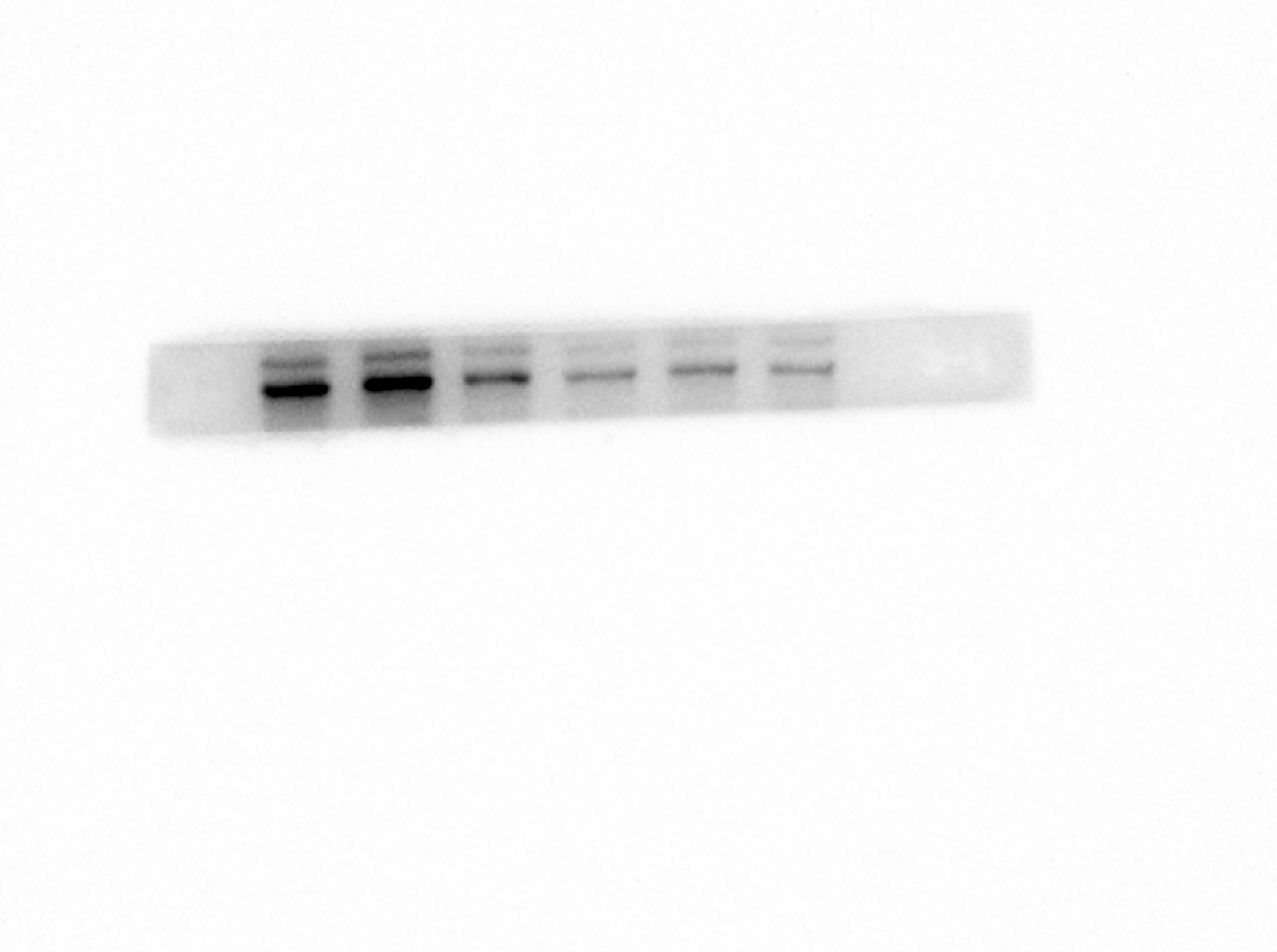

Supplement: Supplementary file 1 [file ijms-25-01206-s001.zip › Original Images for Blots/figure5e/P-ERK1,2(T185,Y187).tif]

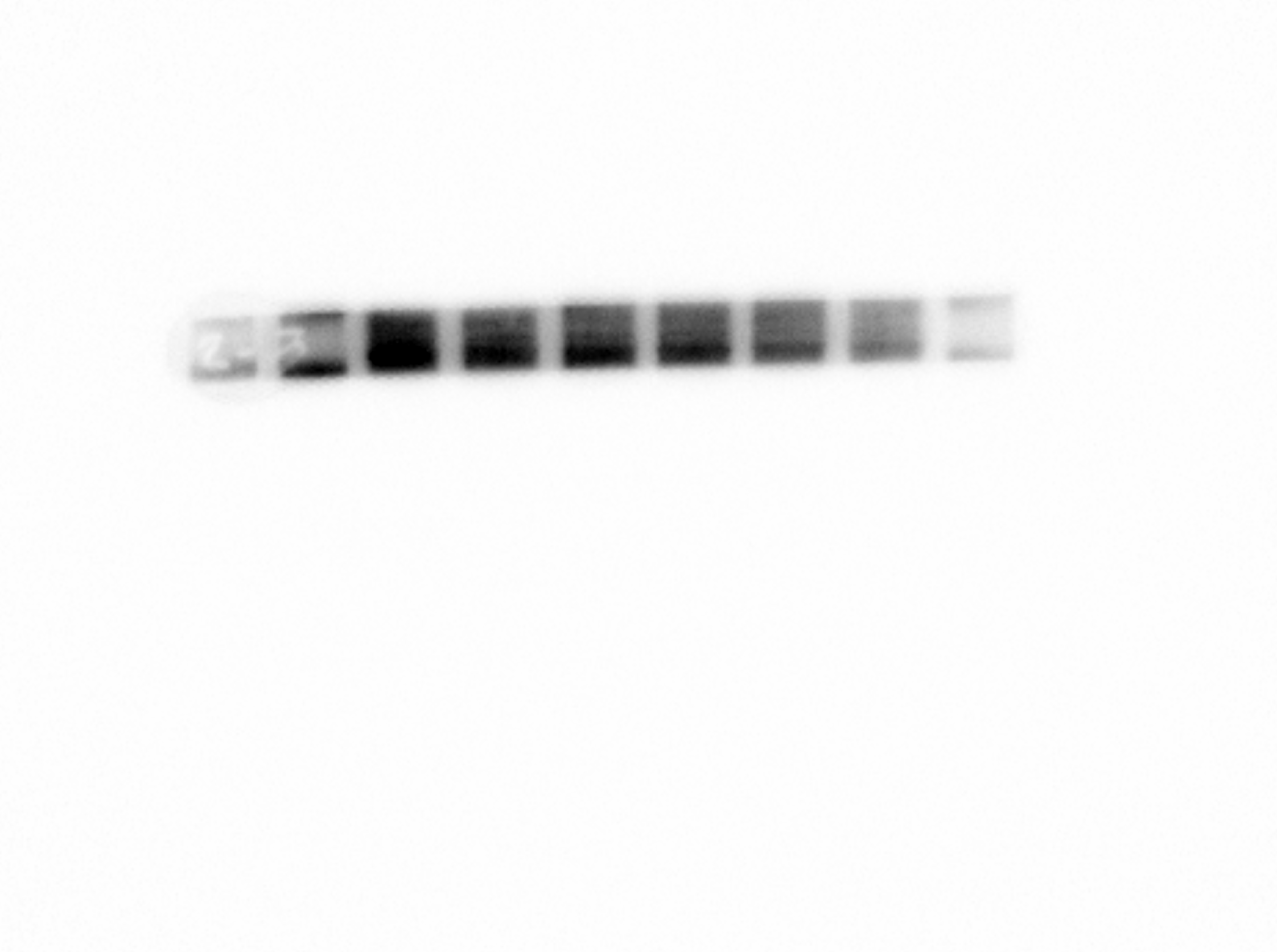

Supplement: Supplementary file 1 [file ijms-25-01206-s001.zip › Original Images for Blots/figure5e/P-P85α-PI3K.tif]

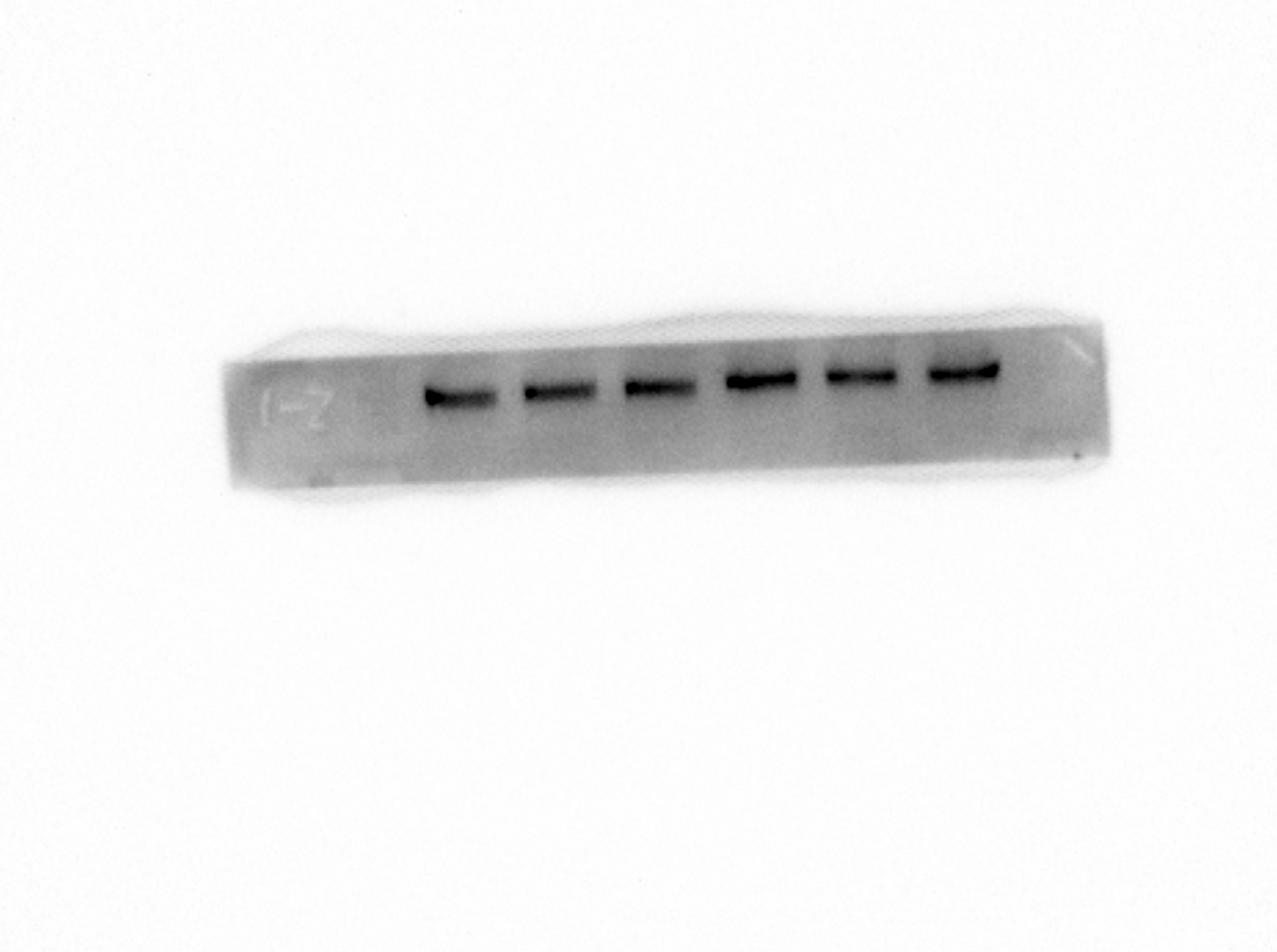

Supplement: Supplementary file 1 [file ijms-25-01206-s001.zip › Original Images for Blots/figure5e/P85α-PI3K.tif]

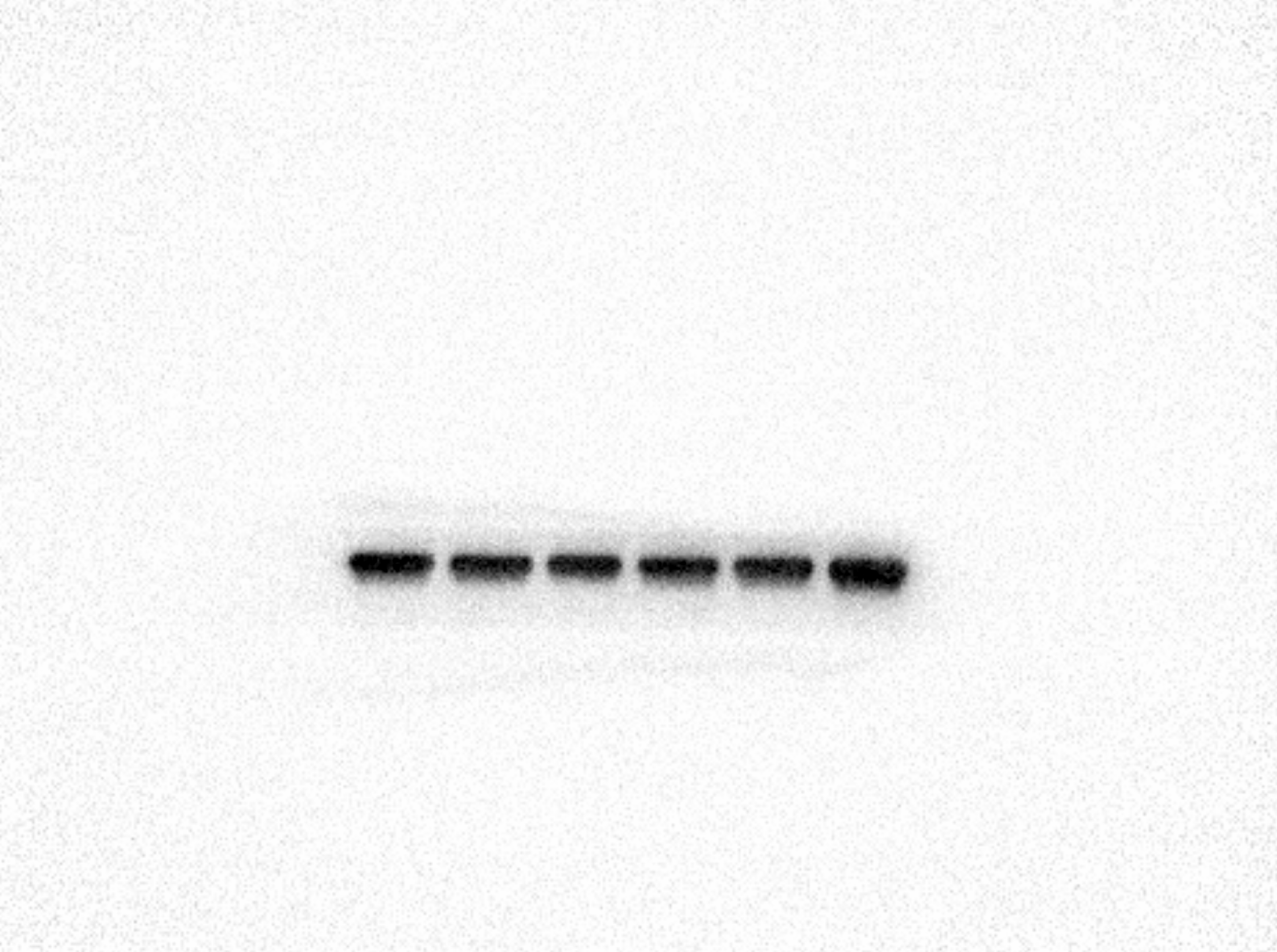

Supplement: Supplementary file 1 [file ijms-25-01206-s001.zip › Original Images for Blots/figure5e/β-actin.tif]
